# Understanding the molecular basis of mesenchymal stem cell stemness: implications for clinical applications

**DOI:** 10.1038/s41419-025-08094-x

**Published:** 2025-11-03

**Authors:** Tong Ming Liu, Wikie Tew, Zheng Yang, Bing Lim, James Hoi Po Hui, Eng Hin Lee, Yuin-Han Loh, Simon Cool

**Affiliations:** 1 Institute of Molecular and Cell Biology (IMCB), Agency for Science Technology and Research (A*STAR), Singapore, Singapore; 2 Department of Orthopaedic Surgery, Yong Loo Lin School of Medicine, National University of Singapore, Singapore, Singapore; 3 NUS Tissue Engineering Program, Life Sciences Institute, National University of Singapore, Singapore, Singapore; 4 Thymmune Therapeutics Inc., Cambridge, MA USA; 5 School of Chemical Engineering, University of Queensland, Brisbane, QLD Australia

**Keywords:** Stem-cell research, Stem cells

## Abstract

Human mesenchymal stem cells (MSCs) have been studied in over 1500 clinical trials to treat over 30 diseases. However, the understanding towards MSC stemness remains under studied. So far, little is known about how MSCs maintain undifferentiated state or commit to specific lineages under different microenvironmental cues. The lack of comprehensive understanding regarding MSC stemness greatly hampers the translation of research findings into successful clinical application due to unclear mechanism of action. Emerging evidence shows that a variety of genetic factors delicately regulate MSC self-renewal and differentiation. In this review, we summarize the role of transcriptional factors, cell cycle regulators, genomic stability genes, cellular quality control, epigenetic regulators, non-coding RNAs, mitochondrial function, growth factors and m6A modification in regulating the stemness of MSCs. Strategies to maintain MSC stemness during ex-vivo expansion are also discussed. This review will deepen understanding of MSC stemness for advancing clinical applications and provide insights into future directions for research aimed at improving MSC-based therapies.

## FACTS

- MSCs are among the most widely used stem cells in clinical applications. However, the molecular mechanisms governing MSC stemness remain poorly understood, significantly impacting their therapeutic potential.
- MSC stemness is finely regulated by transcriptional factors, cell cycle regulators, genomic stability genes, cellular quality control, epigenetic regulators, non-coding RNAs, mitochondrial function, growth factors, and m6A modification.
- Understanding the molecular basis of MSC stemness enhances clinical applications and provides insights for improving MSC-based therapies.

## OPEN QUESTIONS

- What are molecular mechanisms and critical factors of MSC stemness?
- What strategies maintain MSC stemness and enhance the clinical success and commercial viability of MSC-based therapies?
- What is the significance of understanding MSC stemness in applications and regulatory approval?

## Introduction

MSCs are adult stem cells that can differentiate into adipocytes, osteoblasts and chondrocytes. They reside in almost all adult tissues, including bone marrow (BM-MSCs), adipose tissues (AD-MSCs), periosteum (PD-MSCs), Wharton’s jelly (WJ-MSCs), umbilical cord (UC-MSCs), peripheral blood (PB-MSCs), dental pulp (DP-MSCs), and hair follicle (HF-MSCs), among others. Due to their multipotency, immunomodulatory properties, and secretome, MSCs have been widely used for clinical trials. MSCs possess remarkable multilineage differentiation potential, giving rise to osteoblasts, chondrocytes, and adipocytes, which enables their application in the repair of bone, cartilage, and soft tissues. Clinical trials have demonstrated the potential of MSCs in treating osteoarthritis, bone fractures, cartilage defects, and spinal cord injuries. MSCs exhibit immunosuppressive properties by modulating the activity of T cells, B cells, natural killer (NK) cells, and dendritic cells. This makes them highly applicable in the treatment of autoimmune and inflammatory diseases such as graft-versus-host disease (GvHD), systemic lupus erythematosus (SLE), Crohn’s disease, and multiple sclerosis. MSCs have been explored for the treatment of myocardial infarction, ischemic cardiomyopathy, and chronic obstructive pulmonary disease (COPD) by reducing myocardial fibrosis, promoting angiogenesis, and improving cardiac function. MSCs also hold therapeutic potential for pulmonary diseases by modulating the immune response to mitigate inflammation, promoting epithelial repair to restore lung tissue integrity, and reducing fibrosis through the secretion of antifibrotic and regenerative factors. Preclinical and early-phase clinical studies suggest that MSCs may improve outcomes in neurodegenerative and neuroinflammatory conditions. Engineered MSCs have also been utilized as vehicles for targeted drug delivery, particularly in oncology (https://www.clinicaltrials.gov). The therapeutic efficacy of MSCs is largely dependent on the preservation of their fundamental biological properties, commonly referred to as stemness. MSC stemness encompasses their capacity for proliferation, multilineage differentiation into bone, cartilage, and fat. Stemness-retaining MSCs are more effective in promoting regeneration, modulating immune responses, and reducing inflammation. In contrast, loss of stemness reduces these therapeutic benefits. MSCs have a limited life span, leading to cellular senescence and a gradual loss of differentiation potential during expansion. This results in diminished proliferation, impaired differentiation capacity, and elevated secretion of pro-inflammatory cytokines, all of which adversely impact therapeutic efficacy. Although MSCs are immunosuppressive cells, allogeneic MSCs can still trigger immune recognition and rejection over time. This immune pressure may induce functional decline, thereby affecting therapeutic potency. In addition, ensuring consistent MSC quality across batches is a major challenge in clinical application. MSC stemness is affected by culture conditions, passage number, and manufacturing protocols. Rigorous quality control measures are essential to preserve stemness and ensure clinical efficacy. So far, understanding the molecular basis of MSC stemness lags far behind the widespread application of MSCs. Little is known about key transcription factors and gene networks governing MSC stemness, which greatly affect the clinical application of MSCs including approval of MSC products due to unclear mechanisms of action.

**Fig. 1:**
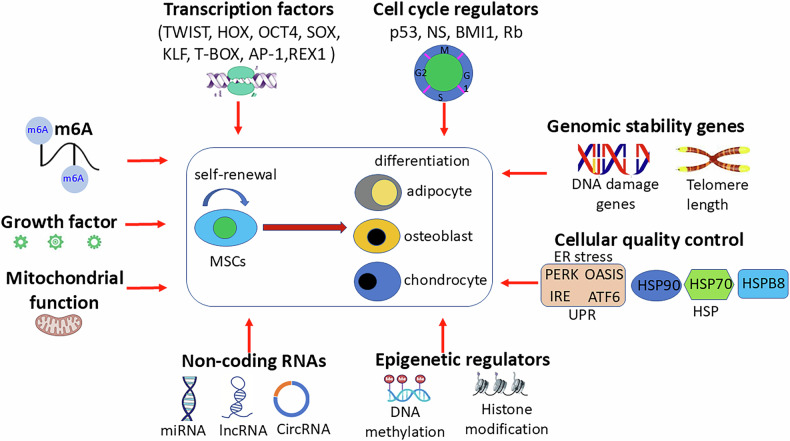
MSC stemness is tightly regulated by various regulators. Genetic regulators that govern MSC stemness include transcription factors, cell cycle regulators, genomic stability genes, cell quality control, epigenetic regulators, non-coding RNAs, mitochondrial function, growth factors, and m6A. Transcription factors, including TWIST, HOX, OCT4, SOX, KLF, T-BOX, AP-1, and REX1, exert their effects by binding to the upstream regulatory elements of target genes. Cell cycle regulators, including p53, NS, BMI1, and Rb, regulate MSC self-renewal and differentiation. Genomic stability genes, such as DNA damage genes and telomere length, are critical to MSC stemness. Cell quality control, including the unfolded protein response (UPR) and the heat shock proteins (HSP), reduces the production of defective proteins by refolding, degrading, or delivering to distinct quality control compartments. Epigenetic regulators, such as DNA methylation and histone modification, modulate the self-renewal and differentiation of MSCS at both chromatin and transcriptional levels without altering the DNA sequences. Non-coding RNA, including miRNAs, long non-coding RNAs (lncRNAs), and circular RNAS (cirRNAs), play important roles in MSC stemness as a functional non-coding RNA molecule. Mitochondrial function plays a critical role in MSC homeostasis and differentiation. Growth factors play an important role in maintaining the stemness of MSCs. m6-methyladenosine (m6A) plays an important role in cell senescence and differentiation of MSCs.

Increasing evidence shows that intrinsic and extrinsic regulators delicately regulate the stemness of MSCs. We have thus made a comprehensive review of various genetic factors, including transcriptional factors, cell cycle genes, genomic stability genes, cellular quality control, epigenetic regulators and non-coding RNAs that regulate the stemness of MSCs (Fig. 1). In addition, the strategies to preserve MSC stemness are also discussed. This review will provide the reader with a brief overview of the genetic regulators regulating MSC stemness, which will provide insights into the molecular basis of MSC stemness and the development of better therapeutic strategies to improve the clinical application of MSCs.

## Genetic regulators governing MSC stemness

### Transcriptional factors regulating MSC stemness

Transcriptional Factors (TFs) are proteins expressed in nuclei, which bind to the upstream regulatory elements of target genes localized in the 5-upstream region to regulate their expression. It was shown that different families of TFs regulate MSC stemness, including self-renewal and differentiation.

### Twist family genes

**Fig. 2:**
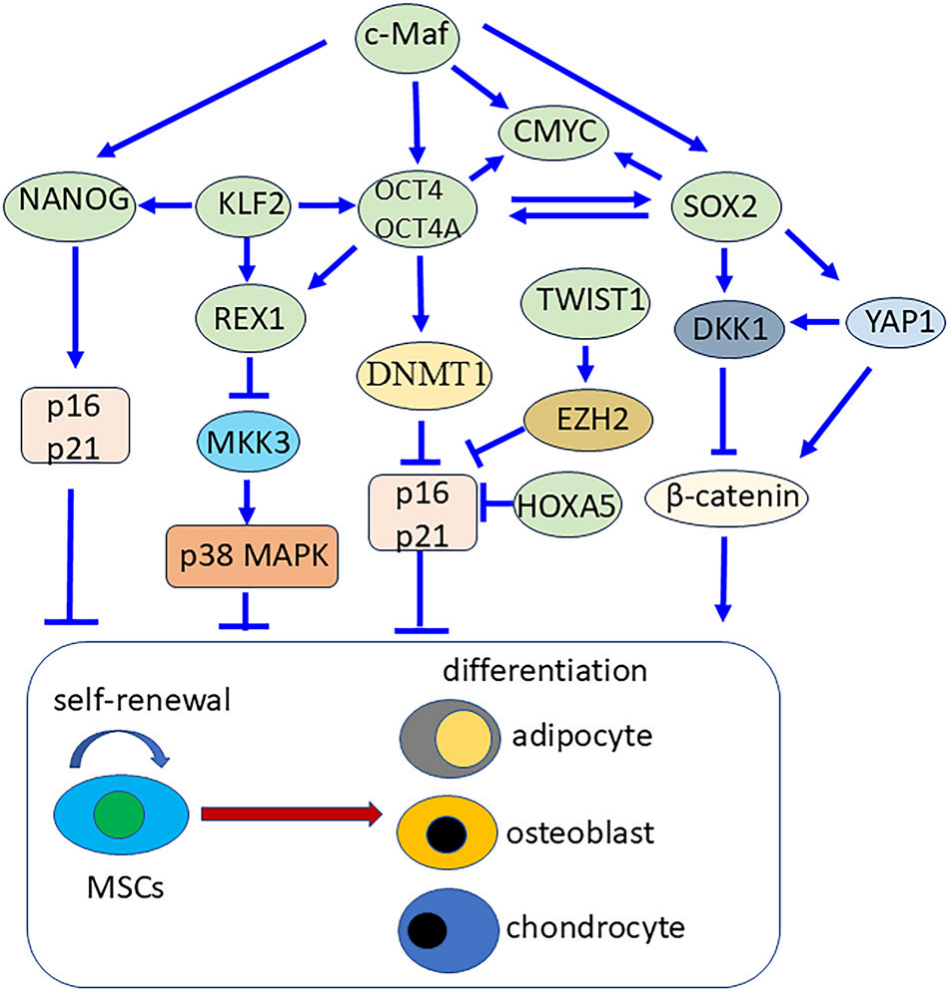
MSC stemness is regulated by an interconnected transcriptional network. c-Maf directly binds to the promoters of OCT4, NANOG, SOX2, and c-MYC to promote MSC stemness. One of its downstream effectors, KLF2, enhances proliferation and maintains the undifferentiated state of MSCs by upregulating pluripotency markers such as OCT4, NANOG, and REX1. REX1, in turn, binds directly to MKK3, an upstream activator of the p38 MAPK pathway, influencing stemness through phosphorylation signaling. OCT4, also downstream of c-Maf, contributes to MSC stemness by regulating DNMT1, thereby repressing p16 expression in cooperation with NANOG. Additionally, the OCT4A isoform supports stemness through downstream targets including REX1, SOX2, and c-MYC. SOX2 directly regulates YAP1, a key effector of the Hippo pathway, and interacts with β-catenin. SOX2 also binds to the promoter of DKK1, a secreted Wnt inhibitor, thereby modulating MSC differentiation via Wnt signaling and senescence via c-MYC. TWIST1, HOXA5, and NANOG suppress the expression of senescence markers, such as p16INK4a and p21CIP1/WAF1 (CDKN1A) to inhibit senescence of MSCs.

The Twist family genes include Twist1 and Twist2 (also called Dermo-1), both of which are part of the basic helix-loop-helix (bHLH) transcription factor family. These proteins contain an N-terminus bHLH domain that binds to DNA at specific sites (E-boxes) and a C-terminus Twist box domain that controls whether genes are turned on or off. Twist1 and Twist2 are highly expressed in MSCs, their levels decrease during in vitro expansion. Overexpression of Twist1 and Twist2 in BM-MSCs increased STRO-1 expression (a stemness marker), promoted proliferation and adipogenesis, but inhibited osteogenesis and chondrogenesis, suggesting they help maintain MSC stemness [1]. Consistently, higher Twist1 expression is linked to stronger MSC proliferation, colony formation (CFU-F), and trilineage differentiation [2]. In contrast, knocking down Twist1 increased senescence in MSCs, altered metabolism (increased oxygen consumption), and triggered a senescence-associated secretory phenotype (SASP) [3]. Mechanistically, Twist1 increases EZH2, which silences senescence genes p14 and p16 by modifying their chromatin via H3K27me3 (Fig. 2). Twist1 also blocks E47, a factor that activates p16 in senescent cells [4]. These findings show that Twist1 and Twist2 regulate MSC stemness, proliferation, metabolism, and senescence, making them key players in MSC function.

### HOX family genes

HOX genes are a family of highly conserved transcription factors that act as master regulators of cell fate, patterning, and differentiation during vertebrate development [5]. In humans, there are 39 HOX genes grouped into four clusters on different chromosomes. In MSCs, the expression pattern of HOX genes, known as the “HOX code”, is stable throughout life and reflects the tissue origin of the MSCs [6]. This code is resistant to changes from external factors and can help distinguish MSCs from different sources, such as bone marrow and cord blood [7]. HOXA5 promoted osteogenic differentiation and proliferation in dental pulp MSCs. Its deletion impaired bone-forming ability and induced cell cycle arrest by upregulating p16^INK4a^ and p18^INK4c^, and downregulating cyclin A [8] (Fig. 2). HOXB7 expression declines with age. Its overexpression enhanced MSC proliferation, reduced aging markers, and improved bone and cartilage differentiation [9, 10]. HOXC10 is found in amnion-derived MSCs but not in most decidua-derived MSCs, making it a potential marker to distinguish MSC subtypes within the same tissue [11]. HOXA11 is expressed in periosteal MSCs and is critical for bone repair. Its expression increased after injury, and its absence impaired bone and cartilage formation [12, 13]. Overall, HOX genes regulate MSC stemness, including their ability to self-renew and differentiate into specific cell types.

### OCT4

The POU genes represent a diverse class of transcription factors, which is distinguished by the N-terminal POU-specific domain (POU_S_) and the C-terminal homeodomain (POU_HD_) tethered by a flexible linker [14, 15]. Octamer-binding transcription factor 4 (Oct4), also known as the POU domain, class 5, transcription factor 1 (POU51), is a well-known TF extensively associated with maintaining ESC pluripotency and somatic cell reprogramming. OCT4 comprises two alternative spliced isoforms, OCT4A and OCT4B [16, 17]. In MSCs, OCT4 expression is highly influenced by serum composition, hypoxia culture, and passage number [18, 19]. OCT4 overexpression promoted the proliferation, CFU-F, and chondrogenesis of MSCs [19, 20]. In human hair follicle MSCs (hHF-MSCs), OCT4 overexpression enhanced cell cycle progression, proliferation, and osteogenesis of hHF-MSCs by upregulating DNMT1 to suppress p21 [21, 22] (Fig. 2). In contrast, OCT4A knockdown reduced proliferation through cyclin A2 (CCNA2) downregulation and inhibited adipogenesis by upregulating DTX1 while decreasing HDAC2 and HDAC4 expression in hUC-MSCs [23]. These indicate a crucial role of OCT4A in maintaining MSC in a stem-like and undifferentiated state. In hAD-MSCS, chromatin immunoprecipitation (ChIP) analysis revealed Argonaute 2 directly regulated OCT4, which targeted Methyl-CpG Binding Domain Protein 6 (MBD6) to regulate genes associated with stemness-associated genes and self-renewal activity of MSCs [24] (Fig. 2). In hBM-MSCs, ChIP analyses demonstrated direct binding of OCT4 to the DNMT1 promoter, which in turn mediates methylation of downstream target genes. This epigenetic modification suppressed expression of cellular senescence markers such as p16 and p21 as well as genes associated with lineage differentiation [19].

### SOX family genes

The SOX family belongs to the high mobility group (HMG) superfamily, characterised by an HMG-box domain and three α-helices HMG-box domains. The SOX family consists of 20 TFs classified into seven subgroups [25]. Emerging evidence indicates that SOX2 also plays an important role in maintaining MSC stemness and suppressing senescence. SOX2 was reduced upon in vitro expansion and could be rescued by low cell density of culture [26]. Senescent MSCs exhibited reduced SOX2 expression alongside increased levels of the senescence markers p16 and p21, suggesting reduced SOX2 is associated with the onset of senescence in MSCs [27, 28]. Knockdown of SOX2 in hUC-MSCs resulted in a decrease of c-MYC, indicating that SOX2 regulates MSC proliferation through c-MYC. ChIP assay and luciferase reporter assay further revealed that SOX2 directly upregulated Dickkopf WNT Signalling Pathway Inhibitor 1 (DKK1), thus antagonizing the WNT signalling pathway, which is a critical switch in enhancing osteogenesis and inhibiting adipogenesis [29]. ChIP assay also identified Yes1 Associated Transcriptional Regulator (YAP1), a Hippo pathway effector, as a target of SOX2 in osteoprogenitor cells. YAP1 was able to induce DKK1 and antagonize Wnt/β-catenin signals, which further inhibited the WNT signalling pathway [30] (Fig. 2). Mechanistically, SOX2 expression is regulated by the PI3K/AKT signaling pathway, and the PI3K/AKT/SOX2 axis modulates the p53/p21/CDK2 pathway, thereby influencing cellular senescence [31]. Apart from SOX2, SOX9, a well-known master regulator of chondrogenesis, also regulates apoptosis, differentiation, and proliferation of MSCs. Sox9 knockdown led to apoptosis of rBM-MSCs by upregulating caspase 3/7 activity and downregulating anti-apoptotic protein Bcl-2. p38 mitogen-activated protein (MAP) kinase was also impaired, resulting in the inhibition of CEPBβ required for adipogenesis and increase in stability of cyclin D1 that induced p21, an inducer of apoptosis and inhibitor of proliferation, and osteocalcin [32]. Knockdown of SOX9 in hUC-MSCs decreased expression of IL-8, stemness-related transcriptional factor OCT4, and Spalt Like Transcription Factor 4 (SALL4), and negatively affected the pro-healing ability in a skin wound model [33]. Endogenous expression of SOX11 decreased with each passage in hBM-MSCs, and knockdown of Sox11 with siRNA reduced the proliferation and osteogenesis of hMSCs, suggesting Sox11 is a positive regulator of MSC stemness [34].

### Krüppel-like factor family genes

The Krüppel-like Factor **(**KLF) family comprises 17 genes in three groups with Cys2His2 zinc finger domain, which binds to the CACCC or GC box DNA elements. KLFs play diverse roles in proliferation, differentiation, growth, development, and survival [35]. In hBM-MSCs, gain- and loss-of-function studies showed KLF2 increased proliferation and maintained an undifferentiated state of MSCs by positively regulating pluripotency markers (OCT4, Nanog, and Rex1) (Fig. 2) and negatively regulating chondrogenic marker cartilage oligomeric matrix protein (COMP) as well as adipogenic marker lipoprotein lipase [36]. KLF2 directly binds to FGFR3 to regulate stemness of hMSCs [37]. Gene profiling showed that KLF4 was overexpressed in hAD-MSCs and hBM-MSCs compared with control fibroblasts. KLF4 decreased during adipogenesis and osteogenesis of MSCs. ChIP assay revealed KLF4 directly bound to the promoter of FZD1 of the WNT signalling pathway and Inhibin beta A (INHBA) of the TGF-β family in undifferentiated MSCs (Fig. 2). KLF4 knockdown improved the lineage differentiation of MSCs, suggesting KLF4 retains undifferentiated state of MSCs [38]. KLF15 was activated during chondrogenic differentiation. KLF15 knockdown slowed down chondrogenesis, whereas KLF15 overexpression improved chondrogenesis by activating the expression of SOX9 [39].

### T-box (TBX) family genes

The TBX family comprises 17 genes and is characterised by T-domain binding to the DNA consensus sequence of TCACACCT. They are further classified into five subfamilies [40]. T-box transcription factors are well-known for their function in several cellular processes such as organogenesis, cell cycle, self-renewal, and differentiation of embryonic and several other adult stem cells [41, 42]. Tbx3 expression decreased during in vitro expansion of MSCs and increased during osteogenesis. Tbx3 knockdown decreased proliferation and slowed down osteogenic differentiation of hADSCs [43], whereas TBX3 overexpression promoted self-renewal, bypassed senescence, and enhance proliferation of MSCs [44]. TBX3 played a key role in regulating cellular senescence by repressing p14^ARF^ expression through interactions with histone deacetylases (HDACs), and by inhibiting p16^INK4a^ either through suppression of cyclin-dependent kinase activity or by cooperating with the polycomb protein EZH2 to promote its epigenetic silencing [45, 46]. However, another study with Tbx20 overexpression inhibited osteogenesis of AD-MSCs with decreased osteogenic genes, Alizarin Red S staining for calcium deposit, and ALP activity [47]. These studies implicate the role of Tbx in proliferation and osteogenic differentiation in MSCs. Whether other members of the T-box family regulate MSC stemness remains to be elucidated.

### AP-1 family transcription factors

The AP-1 family comprises four subfamilies of TFs, including Fos, Jun, musculoaponeurotic fibrosarcoma (Maf), and activating transcription factor (ATF). They are characterised by the basic leucine-zipper (bZIP) domain, which dimerizes with another adjacent basic domain and forms the scissor-shaped α-helical structure. One of the identified AP-1 TFs regulating MSC stemness is c-Maf, which belongs to the Maf subfamily. Reduced c-Maf led to decreased proliferation and osteogenic differentiation during ROS-induced senescence. ChIP assay showed that c-Maf directly bound to the promoters of OCT4, Nanog, Sox2, c-MYC, KLF4, and CCND2 (Fig. 2), showing that c-Maf acts as a regulator of proliferation and pluripotency of hAD-MSCs [48].

### REX1

REX1 is a known marker of pluripotency, which involves ES cell self-renewal and the reprogramming of X-chromosome inactivation during the acquisition of pluripotency. hUC-MSCs and hAD-MSCs express higher REX1 and lower p38 MAPK, whereas BM-MSCs are opposite. REX1 knockdown decreased cell proliferation and osteogenesis but increased adipogenesis and the phosphorylation of p38 MAPK. The p38 MAPK inhibitor could rescue decreased cell growth and expression of CDK2 and CCND1 by knockdown of REX1 in hUCB-MSCs. ChIP assay showed REX1 directly bound to MKK3, an upstream regulator of p38 MAPK, to exert its effects on MSC stemness [49] (Fig. 2).

## Role of cell cycle regulators

The cell cycle comprises three distinct phases. The quiescent state (G0) (resting phase), interphase (G1, S, G2), and mitosis phase (M). Negative cell cycle regulators, such as the tumour suppressor proteins and CDK inhibitors (CDKIs), result in cell cycle arrest, leading to quiescence or senescence. Quiescence is reversible and occurs at the G0 phase, typically mediated by cyclin D1 and p27-dependent CDK inactivation, whereas senescence is irreversible and occurs mainly at the G1 and G2 phases. Positive regulators, including the oncogenes and CDK-cyclin complexes, drive the cell cycle forward to allow cell proliferation. They can balance cell cycle arrest and progression, therefore meeting the regeneration needs by preventing the exhaustion of the stem cell pool and malignant transformation. It was shown that cell cycle-associated genes such as p53, Nucleostemin, BMI1, and Retinoblastoma regulate MSC self-renewal and differentiation.

### p53

p53, also dubbed the “guardian of the genome”, is a regulator of apoptosis, DNA repair, and cell cycle arrest. As a tumour suppressor protein, p53 halts the cell cycle by inducing the expression of cell cycle arrest genes such as p21, 14-3-3σ, wild-type p53-induced phosphatase 1 (WIP1), and Growth Arrest and DNA Damage-inducible 45 (GADD45), while suppressing the expression of cell cycle progression genes such as cyclin B1 [50]. In the specific context of MSCs, p53 knockout MSCs displayed morphologic and phenotypic changes, increased proliferation rate, shortened doubling time, and promoted CFU-F formation. In addition, p53 knockout also altered MSC differentiation, genomic stability, c-Myc level, and anchorage-dependent growth [51]. Consistent with these findings, p53 depletion increased proliferation and CFU-F of MSCs by increasing expression of TWIST2, improved osteogenesis at the expense of adipogenesis by suppressing PPARG, and diminishing mitochondrial ROS production, suggesting p53 is a negative regulator of self-renewal and osteogenesis of MSCs [52].

### Nucleostemin (NS)

NS is a novel p53-binding nucleolar protein enriched in stem cells. As a multiplex regulator of cell-cycle progression. It may interact with p53 to maintain the proliferation of stem cells by stabilizing MDM2 to prevent its ubiquitination and proteasomal degradation. Aberrant levels of NS can trigger p53-dependent cell cycle arrest. NS can directly interact with and inhibit mouse double minute 2 homolog (MDM2), an E3 ligase that tags p53 for degradation at a high level. NS also promoted ribosomal proteins L5 and L11 to indirectly degrade MDM2 at a low level [53]. This study partially explains NS localization at the nucleolus, where rRNA transcription and assembly occur, and how it regulates cell proliferation through ribosome synthesis [54].

The functional role of NS is investigated mainly in neuronal stem cells and cancer cells. NS is also a marker of proliferating MSCs [54–56]. NS was relatively highly expressed in undifferentiated BM-MSCs but declined sharply upon induction of differentiation, becoming undetectable within 6 h of initiation [55]. NS decreased during the differentiation of MSCs into adipocytes, osteocytes, and chondrocytes. FGF2 increased MSC proliferation dose-dependently, which could be abolished by NS knockdown [54]. NS localizes along chromosome arms; however, disruption of mitotic microtubules leads to its dissociation from the chromosomes and dispersion throughout the cytoplasm. Additionally, NS mRNA expression gradually declines in aging UC-MSCs and progressively disappears during MSC adipogenic differentiation [56]. These findings suggest that NS serves as a marker for human MSCs, reflecting their proliferation and differentiation states. Mechanistically, NS knockdown significantly decreased the proliferation of rBM-MSCs in parallel to reduced survivin and cyclin D1, two well-known regulators of cell proliferation. There was no noticeable change in the expression level of p21, the main effector of p53, suggesting NS-regulated MSC proliferation independently of p53 [57].

### BMI1

The epigenetic regulator BMI1, also known as RING finger protein 51 (RNF51) or Polycomb group RING finger protein 4 (PCGF4), is a key component of the Polycomb group complex 1 (PRC1). BMI1 promotes cell cycle progression and regulates senescence by acting as a transcriptional repressor at the *INK4a/ARF* locus [58]. BMI1 regulated proliferation and apoptosis of MSCs via repressing p16^INK4a^ expression. BMI1 also regulated the osteogenesis and adipogenesis of MSCs [59]. BMI1 inhibited p16, p19, and p27 to maintain the self-renewal of BM-MSCs. BMI1 knockout reduced chondrocyte proliferation and increased apoptosis; consequently, BMI1-deficient mice exhibited skeletal growth retardation, which was partly attributed to a shift in adipogenic-osteogenic differentiation, at least in part through the upregulation of SIRT1 expression [60]. BMI1 knockout mice also exhibited intervertebral disc degeneration, accompanied by a significant reduction in antioxidant enzymes, including superoxide dismutases (SOD1 and SOD2) and glutathione peroxidases (GPX1 and GPX3), as well as downregulation of cell cycle regulators p16, p21, and p53. Notably, this phenotype could be ameliorated by antioxidant treatment with N-acetylcysteine (NAC). Hypoxic culture enhanced the proliferation and immunosuppressive effects of hUC-MSCs by upregulating BMI1; these effects were abolished by BMI1 knockdown and accompanied by a reduction in COX-2/PGE2 expression. In contrast, BMI1 overexpression increased phosphorylated p38 MAP kinase. Mechanistically, BMI1 directly bound to the promoter of MAPK phosphatase 1 (MKP-1, also known as DUSP1) to suppress its expression. These data suggested that BMI1 regulates the immunomodulation of hUCB-MSCs through p38 MAP kinase-mediated COX-2 expression [61]. MCP-1, also known as CCL2, is secreted major component of senescence-associated secretory phenotype (SASP) during UCB-MSC senescence, which was directly bound by BMI1 to its regulatory element to repress its expression [62].

### Retinoblastoma

Retinoblastoma (Rb) belongs to the pocket protein family, which serves as a negative regulator of cell growth and acts primarily at the G0 and early G1 phase by inhibiting E2F transcription factors and preventing the transcription of its proliferation-associated target genes [63]. RB1 plays essential roles in MSC self-renewal and differentiation. RB1 knockdown increased p53 and p21; therefore MSCs lacking RB1 displayed a senescent phenotype with impaired self-renewal properties and decreased differentiation potential [64]. RB is expressed higher in the early passage of MSCs with higher DNMT1 than late passage of MSCs. RB knockdown decreased the proliferation and differentiation potentials, induced premature senescence of MSCs at early passage. In contrast, RB overexpression prevented senescence and enhanced differentiation potentials of MSCs at late passage. Similarly, DNMT1 knockdown promoted senescence and decreased differentiation potentials of MSCs at early passage, whereas DNMT1 overexpression has the opposite effect at late-passage MSCs, suggesting that RB preserves the proliferation and differentiation potential of MSCs by upregulating DNMT1 [65]. Downregulation of RB1 and S18-2 was correlated with decreased expression of stemness-related genes, such as OCT-4, KLF-4, NANOG and c-MYC in human MSCs [66].

## Genome stability genes safeguarding MSC genomic integrity

### DNA damage genes

Genomic stability is also critical to MSC stemness, because MSCs give rise to progenies that regenerate and repair the tissues in our body. Adult stem cells are mainly in a quiescence state and are constantly exposed to DNA-damaging agents. In contrast to most terminally differentiated somatic cells, adult stem cells undergo extensive proliferation and differentiation processes, increasing the likelihood of DNA damage during DNA replication. Unfaithful DNA damage repair leads to the depletion of the stem cell pool by triggering apoptosis, senescence, and differentiation of stem cells that can carry mutations to their progenies [67]. As adult stem cells, MSCs gradually lose the self-renewal and differentiation potential during in vitro expansion, which was associated with the activated DNA damage response pathway and the accumulated DNA damage. MSCs accumulate DNA damage during in vitro expansion, which leads to a significant decrease in the differentiation potential of MSCs [68]. Expanded MSCs at P3 were inferior to freshly isolated bone marrow mononuclear cells in chondrogenesis and cartilage repair, in parallel with decreased telomerase activity, changed chromosomal morphology, downregulated cell cycle, DNA replication and mismatch repair (MMR) pathways [69]. Long-term expansion of mMSCs led to a gradual loss of their ability to recognize double-strand DNA breaks, resulting in impaired DNA damage response and increased chromosomal instability, as evidenced by a higher number of micronuclei [70].

### Telomere length

The telomere is a specific DNA–protein structures with repetitive DNA sequences at the end of a chromosome, which protects the genome from degradation and maintains self-renewal and the undifferentiated state of stem cells. Human telomerase reverse transcriptase (hTERT) prolonged the lifespan and maintained the osteogenic potential of hMSCs. Upon xenogenic transplant, hTERT-expressing hMSCs formed more bone tissues [71, 72]. On the contrary, telomerase knockout MSCs lose the capability to differentiate into adipocytes and chondrocytes even at early passages. Telomerase-depleted MSCs underwent senescence at late passages, exhibiting telomere shortening and chromosomal end-to-end fusions. These results suggest telomerase regulates the differentiation of MSCs [73]. A combination of serum deprivation and aphidicolin increased the expression of hTERT mRNA and protein. This led to synchronized hMSCs at S phase, showing cell cycle-dependent telomerase expression in hMSCs [74]. hTERT knockdown decreased cell proliferation and increased the senescence and apoptosis of MSCs, whereas the hTERT overexpression had opposite effects on MSCs via PI3K/AKT signalling pathway [75]. It was consistent that increased telomerase activity by Taurine improved chondrogenesis in DP-MSCs [76]. These studies indicate that telomerase plays a crucial role in MSC self-renewal and differentiation.

Accumulating evidence suggests that a subset of MSCs may activate alternative lengthening of telomeres (ALT), a telomerase-independent mechanism that relies on homologous recombination-based processes to elongate telomeres. This results in highly heterogeneous telomere lengths. ALT activation is more commonly observed in immortalized or transformed MSCs lacking detectable telomerase activity. Notably, cells utilizing ALT often exhibit a wide range of telomeric variant repeats, which vary significantly between cell lines [77]. ALT-mediated telomere maintenance has the potential to extend MSC lifespan without reactivating telomerase, potentially lowering the oncogenic risk compared to hTERT overexpression. However, ALT activity is frequently associated with genomic instability, raising safety concerns for therapeutic applications. While ALT activation is rare in primary MSCs, its possible emergence under long-term culture or cellular stress highlights the importance of closely monitoring telomere dynamics and genomic integrity in MSC-based therapies. Further research is needed to elucidate the molecular triggers, prevalence, and functional consequences of ALT activation in MSC populations.

## Cellular quality control mechanisms influencing MSC stemness

Cellular quality control, including the unfolded protein response and the heat shock proteins, reduces production of defective proteins by refolding, degrading, or delivering to distinct quality control compartments.

### The unfolded protein response

The unfolded protein response (UPR) is an adaptive reaction to maintain cell function and viability by controlling protein homeostasis of endoplasmic reticulum (ER) and decreasing unfolded protein load. The UPR increases cell survival under ER stress conditions as an evolutionarily conserved adaptive mechanism [78]. The UPR is not activated under normal conditions. The UPR can be activated under certain stresses, such as altered glycosylation or hypoxia. UPR is activated by the accumulation of misfolded or unfolded proteins and helps maintain cellular homeostasis and survival under ER stress. Correct folded proteins are translated to the Golgi apparatus during protein processing, whereas unfolded or misfolded proteins are accumulated in the ER lumen to be targeted or refolded for degradation. When UPR cannot alleviate ER stress, the stressed cells will be self-destructed through apoptosis. Exogenous ROS, such as Hydrogen peroxide, induced apoptosis of MSCs via both mitochondrial pathways and ER by activating p38 and JNK. The former regulated early apoptosis of MSCs, while the latter was involved the late apoptosis [79]. Tunicamycin (TM) significantly upregulated the expression of ER chaperones GRP78 and GRP94 in MSCs, aiding the cells in resisting stress-induced injury [80]. Free fatty acid palmitate induced ER stress and apoptosis in hBM-MSCs by activating p38, which could be prevented by an AMPK activator [81]. C/EBPβ upregulated ER stress, as its overexpression in MSCs induced ER stress, evidenced by increased expression of the pro-apoptotic marker CHOP and decreased expression of the chaperone gene BiP [82]. Arsenic trioxide induced mitochondria-modulated apoptosis and ER stress in UC-MSCs and BM-MSCs [83]. A significant decline in proteasome activity in hWJ-MSCs was associated with cellular senescence. Overexpression of the β5 catalytic subunit enhanced proteasome function, leading to increased WJ-MSC proliferation, improved trilineage differentiation potential, and upregulation of stemness markers including OCT4, SOX2, and NANOG [84]. GRP78 is an essential molecular chaperone responsible that binds to misfolded, or unfolded proteins during ER stress. GRP78 overexpression protected against ER stress–induced apoptosis and reduced apoptosis during BMP2-induced chondrogenesis, whereas GRP78 knockdown activated the ER stress-specific caspase cascade in developing chondrocytes [85].

**Fig. 3:**
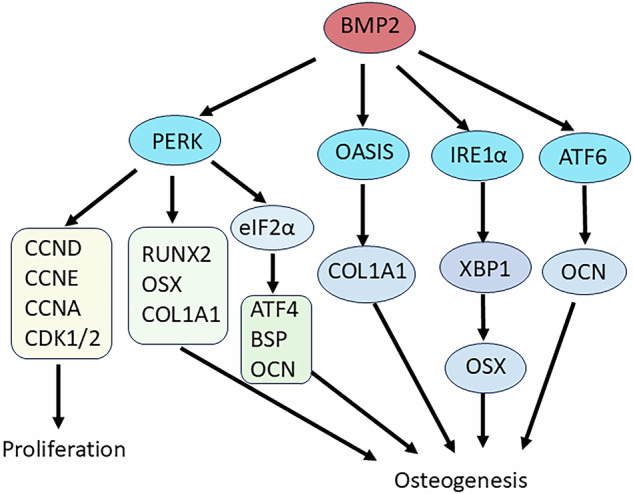
BMP2 induces osteogenesis of MSCs via the unfolded protein response (UPR) transducers. BMP2 induces osteogenesis of MSCs by activating the unfolded protein response (UPR) transducers, including PERK, OASIS, IRE1α and ATF6. PERK regulates master regulator of osteogenesis such as RUNX2 and OSX as well as cell cycle-related genes to regulate osteogenesis. PERK-eIF2α-ATF4 signaling pathway regulates osteoblastic differentiation by endoplasmic reticulum stress (ERS). OASIS affects the activity of osteoblasts and bone formation by activating COL1A1. IRE1α-XBP1 signaling regulates osteogenesis by promoting OSX transcription. BMP2 induces osteogenesis via RUNX2-dependent ATF6, which directly regulates OCN transcription.

Physiological ER stress results in the maturation of MSCs. BMP2 activates UPR transducers, such as PERK, OASIS, IRE1α, and ATF6, which induced osteogenesis of MSCs [86, 87]. UPR signaling influences bone biology through the PERK-eIF2α-ATF4 pathway. PERK is one of the primary transducers of the UPR signaling pathway. PERK deficiency led to severe osteopenia, decreased mature osteoblasts, secretion of Type-I collagen, and osteoblast differentiation in the bone matrix. PERK deficiency reduced the expression of RUNX2 and Osterix, master regulators of osteogenesis as well as important cell cycle factors, such as CCND, CCNE, CCNA, CDK1, and CDK2 leading to decreased cell proliferation [88] (Fig. 3). Overexpression of PERK enhanced eIF2α phosphorylation and induced ATF4, BSP and OCN expression, showing The PERK-eIF2α-ATF4 signaling pathway regulates osteoblastic differentiation in response to endoplasmic reticulum stress (ERS). ATF4 plays a crucial role in osteogenesis and bone formation as a downstream target of PERK [78] (Fig. 3). OASIS belongs to the CREB/ATF family, which is an ER membrane-bound bZIP (basic leucine zipper) transcription factor with a transmembrane domain. OASIS is highly expressed in osteoblasts and processed by regulated intramembrane proteolysis (RIP) in response to mild ER stress. OASIS is involved in ER-mediated bone formation. OASIS knockout mice displayed abnormally expanded rough ER and severe osteopenia due to reduced COL1A1 in the bone matrix and decreased activity of osteoblasts by targeting and activating COL1A1 [89]. IRE1α is a key mediator of UPR, and its downstream transcription factor, X-box binding protein 1 (XBP1), is essential for BMP2-induced osteogenesis. XBP1 directly targeted OSX, the key TF of osteogenesis. The IRE1α-XBP1 pathway contributed to osteogenesis by promoting OSX transcription, showing that the IRE1α-XBP1 pathway regulates osteogenesis through OSX transcription [90]. In addition, IRE1α inhibited GEP-mediated chondrocyte differentiation as a negative regulator, which was accompanied by altering IHH and PTHrP [91]. BMP2 induced osteogenesis and directly regulated OC transcription through Runx2-dependent ATF6, a UPR transducer [86] (Fig. 3). ATF6 positively regulated endochondral bone formation and chondrocyte hypertrophy via Runx2 [92].

### The heat shock proteins

Heat shock proteins (HSPs) serve as important quality control, which act as molecular chaperons by facilitating the synthesis, folding, re-folding, translocation, stabilization and degradation of proteins. Under harsh conditions such as elevated temperature, oxidative stress and nutrient deprivation, heat shock factors (HSFs) respond to proteotoxic stress by binding to heat shock elements (HSEs), thereby upregulating the expression of heat shock proteins (HSPs). HSPs have been shown to regulate MSC self-renewal and differentiation. HSP90 enhanced MSC viability and protected MSCs from apoptosis induced by serum deprivation and hypoxia by downregulating membrane TLR-4 and ErbB2 receptors to activate their downstream PI3K/Akt and ERK1/2 pathways [93]. Periodic mild heating enhanced osteogenesis and chondrogenesis of hMSCs by significantly upregulating heat shock protein 70 (HSP70). HSP70 knockdown slowed down osteogenesis and chondrogenesis of hMSCs, showing that downregulation of HSP70 impaired the osteogenic and chondrogenic differentiation of hMSCs [94]. Late passage of DP-MSCs displayed decreased osteogenic potential with reduced expression of HSPB8. HSPB8 knockdown abolished osteogenesis of the DP-MSCs, suggesting HSPB8 is essential for the differentiation potential of DP-MSCs [95].

## Epigentic regulators

Epigenetic modification, including DNA methylation, histone modification and m6A, modulates the self-renewal and differentiation of MSCs at both the chromatin and transcriptional levels without altering the DNA sequences, which is a crucial mechanism influencing the stemness of MSCs.

### DNA methylation

One molecular hallmark of epigenetic architecture is DNA methylation. A quintessential methylation status of MSCs is that the expression of stemness-associated genes should be hypomethylated. In contrast, lineage-specific genes are typically hypermethylated but retain the plasticity to become hypomethylated upon induction. Methylome analyses have revealed changes in MSC global DNA methylation landscape during aging and senescence wherein multipotential and proliferation are being compromised [96, 97], suggesting that DNA methylation and demethylation are critical processes to govern the gene expression that contributes to MSC behavior. Global DNA methylation was inhibited by DNMTs inhibitors, such as RG108 (inhibits DNMT1), 5-azacytidine or decitabine (5-aza-2ˊ-deoxycytidine; inhibits DNMT1, DNMT3b), which resulted in an open chromatin architecture to maintain the stemness of MSCs. RG108 is a DNA methyltransferase inhibitor, that induces epigenetic changes at both gene and global-specific levels. In BM-MSCs, RG108 promoted epigenetic activation of NANOG and OCT4 and therefore increased mesenchymal cell markers of CD105, NANOG, and OCT4 through epigenetic modification [98]. RG108 treatment also upregulated anti-senescence genes (TERT, bFGF) and the anti-apoptotic gene (BCL2), while downregulating the pro-apoptotic gene (BAX), thereby protecting MSCs from apoptosis [99].

### Histone modification

Histones modification, such as methylation, demethylation, acetylation and deacetylation, remodels the chromatin architecture which dictates histones either loosening or tightening their grip onto DNA to regulate MSC self-renewal, senescence and differentiation potential.

### Histone methylation

Emerging evidence reveals that the stemness of MSCs is regulated by epigenetic methylation of histone. Zeste homolog 2 (EZH2) is a histone methyltransferase, its expression increased during adipogenesis but decreased during osteogenesis of hMSCs. EZH2 overexpression promoted adipogenesis but inhibited osteogenesis whereas EZH2 knockdown had opposite effects by altering H3K27me3 [100]. EZH2 inhibitor enhanced bone formation and mitigated bone loss [101]. EZH2 knockout MSCs exhibited enhanced trilineage differentiation potential compared with wild-type MSCs. EZH2 knockout mice had thinner cortical bone, leading to decreased mechanical strength due to increased osteoclastogenic potential and upregulated Rankl and M-csf expression [102]. Mechanistically, EZH2 is a downstream target of TWIST1, which inhibited the Ink4A/Arf locus via H3K27me3 to repress transcription of p16/p14 and senescence of human MSCs [4]. In addition, EZH2 is the catalytic subunit of polycomb repressive complex 2 (PRC2), which represses gene transcription via targeting lysine-27 of histone H3. EZH2 was directly regulated by β-catenin [103]. Coactivator Associated Arginine Methyltransferase 1 (CARM1) directly bound to Discoidin Domain Receptor 2 (DDR2) to regulate the expression of DDR2 and MSC senescence via H3R17 methylation [104]. SET domain-containing Protein 4 (SETD4) is another histone lysine methyltransferase, which changes the overall methylation of the MSC genome. SETD4 depletion significantly increased the proliferation of BM-MSCs while reducing their migration and differentiation potential [105].

### Histone demethylation

Demethylation also affects MSC stemness. KDM3A and KDM4C are two H3K9 demethylases, which inversely correlate with MSC aging by regulating NCAPG2 and NCAPD2. KDM3A or KDM4C suppression resulted in robust DNA damage response and aggravated cellular senescence, whereas KDM3A or KDM4C overexpression promoted heterochromatin reorganization and decreased DNA damage response [106]. KDM4A promoted adipogenesis and inhibited osteogenesis of BM-MSCs [107]. KDM5A inhibited osteogenesis by repressing Runx2 expression via H3K4me3 [108], whereas KDM6A inhibited adipogenesis and promoted osteo/chondrogenesis of BM-MSCs [100]. KDM7A was upregulated during adipogenesis and osteogenesis of MSCs. KDM7A knockdown blocked adipogenesis but promoted osteogenic differentiation, whereas KDM7A overexpression exerted opposite effects by directly binding to the promoters of C/EBPα and SFRP1, demethylating both H3K9me2 and H3K27me2 [109].

### Histone acetylation

Histone acetylation via histone acetyltransferases (HATs) is a critical epigenetic modification that neutralises the lysine residues of histone proteins to open the chromatin structure and regulates gene expression, which can be reversed by histone deacetylases (HDACs) to remove the acetyl groups. Histone acetylation has been shown to play a vital role in the self-renewal and differentiation of MSCs. Histone acetyltransferase p300 decreased in senescent hUC-MSCs at late passage, p300 inhibition induced premature senescence and decreased proliferation of hUC-MSCs [110]. Lysine Acetyltransferase 6A (KAT6A) decreased in old MSCs, coinciding with reduced proliferation, colony formation, and osteogenesis of MSCs by regulating antioxidant gene Nrf2 to inhibit ROS accumulation [111]. Nucleosome assembly protein 1-like 2 (NAP1L2), a histone chaperone, was identified to correlate with BM-MSC senescence by comparing the gene expression profiles of human BM-MSCs from young and old donors. NAP1L2 increased in senescent MSCs, which suppressed osteogenic differentiation of MSCs by recruiting SIRT1 to deacetylate H3K14ac on promoters of osteogenic genes. Senescent phenotypes of BM-MSCs could be alleviated by nicotinamide mononucleotide (NMN) via a possible bond between NAP1L2 and NMN [112].

### Histone deacetylation

Histone acetylation influences gene transcription and is regulated by histone deacetylases (HDACs). Aged BM-MSCs exhibit reduced differentiation and proliferation alongside increased senescence, processes mediated by HDACs and longevity-related deacetylases such as sirtuins (SIRT). Sirtuins are NAD^+^-dependent deacetylases that chemically reverse acetyllysine modifications of cellular proteins. In BM-MSCs, SIRT1 knockdown reduced SOX2 protein levels by promoting its acetylation, ubiquitination, and nuclear export, leading to proteasomal degradation and a decline in the self-renewal and differentiation capacities of BM-MSCs. Resveratrol, a known activator of SIRT1, reduced TSA-mediated acetylation and ubiquitination of SOX2, thereby enhancing the colony-forming ability as well as osteogenic and adipogenic differentiation of BM-MSCs in a dose-dependent manner. These suggest that the SIRT1-SOX2 axis is essential for self-renewal capability and multipotency of BM-MSCs by post-translational modification [113]. SIRT1 decreases in aged MSCs, paralleled by cellular senescence and impaired proliferation capability. SIRT1 knockdown led to cellular senescence and significantly decreased cell proliferation in young MSCs, whereas SIRT1 overexpression rejuvenated the senescence phenotype and promoted cell proliferation of aged MSCs. Mechanistically, SIRT1 negatively regulates p16 and p21, essential in driving senescence in response to DNA damage. SIRT1 positively regulates the activity and expression of telomerase instead of telomere length via tripeptidylpeptidase1(TPP1), which protects chromosome ends from DNA damage [114]. SIRT3 is the major mitochondrial deacetylase which reduces oxidative. SIRT3 decreased with age but increased with MSC differentiation. SIRT3 knockdown slowed down adipogenesis and osteogenesis of MSCs, whereas SIRT3 overexpression decreased aging-related senescence and oxidative stress, but enhanced the differentiation potential of late-passage MSCs [115]. SIRT7 knockout displayed severe osteopenia by regulating lysine acylation of SP7/Osterix to decrease bone formation and increase osteoclasts [116].

### m6A regulating MSC stemness

m6-methyladenosine is the most prevalent modification in coding and noncoding RNAs of the eukaryotic transcriptome, which is a common and reversible RNA modification that plays a crucial role in senescence and differentiation of MSCs. m6A modification is delicately regulated by methyltransferases/writers that write the m6A modification, demethylases/erasers that remove the m6A modification, and m6A readers that govern the function of m6A modification. m6A modification occurs across nearly the entire transcriptome and regulates RNA splicing, stability, decay, transport, and translation. METTL3, an m6A methyltransferase, decreases during osteogenesis of BM-MSCs and plays a crucial role in regulating MSC differentiation and survival through m⁶A-dependent mechanisms. METTL3 knockdown decreased the expression of osteogenic genes and formation of mineralized nodules via Akt phosphorylation [117]. METTL3 knockdown greatly reduced the total m6A levels in mRNA and promoted adipogenesis of porcine BM-MSCs via JAK/STAT pathway in a m6A-YTHDF2-dependent manner [118]. METTL3 knockout in BM-MSCs increased bone loss and led to osteoporosis in mice, demonstrating the pathological outcomes of m6A in skeletal diseases [119]. METTL3-mediated m⁶A modification of circSTAT6 promoted the osteogenic differentiation of BM-MSCs by modulating the miR-188-3p/Beclin1 axis [120]. Similarly, METTL3 overexpression enhanced osteogenesis in MSCs from patients with congenital pseudarthrosis of the tibia by stabilizing HOXD8 mRNA via m⁶A modification [121]. In UC-MSCs, METTL3 overexpression facilitated osteogenesis by increasing m⁶A modification of circCTTN [122] and mediating methylation of lncRNA MIR99AHG [123]. Moreover, METTL3 protected BM-MSCs from hypoxia-induced apoptosis by enhancing the mRNA stability of Bcl-2, Mcl-1, and BIRC5, through IGF2BP2 binding in an m⁶A-dependent manner [122, 124]. METTL3‑mediated m6A RNA methylation promoted the differentiation of lung-derived MSCs into myofibroblasts [125]. In BM-MSCs, METTL14 regulated osteogenic differentiation via the m⁶A/IGF2BPs/Beclin-1 signaling axis [126], while METTL16 suppressed osteogenesis by promoting m⁶A modification of PPARγ transcripts, thereby enhancing its expression [127]. Additionally, METTL3 enhanced chondrogenic differentiation of synovium-derived MSCs by targeting MMP3, MMP13, and GATA3 [128]. The m6A modification can be reversed by m6A demethylases/erasers, such as FTO and ALKBH5, which belong to family of iron (II)/a-ketoglutarate (a-KG)-dependent dioxygenases recognizing cytosine and adenine methylation in DNA and RNA. ALKBH5 is one of the key m6A demethylases, which regulates MSC senescence via m6A modification. The m6A modifications increased while ALKBH5 decreased during BM-MSC senescence. In BM-MSCs, ALKBH5 normally functions to remove m6A modifications from RNA. Knockdown of ALKBH5 led to the accumulation of m6A marks on target mRNAs. Specifically, increased m6A modification on CYP1B1 mRNA enhanced its stability, resulting in elevated CYP1B1 protein expression and thereby accelerating MSC senescence [129]. In contrast, in AD-MSCs, m6A modifications decrease, while ALKBH5 expression increases during aging. In this context, ALKBH5 regulated the expression of a different target, CDKN1C, a cyclin-dependent kinase inhibitor that enforced cell cycle arrest, by modulating its mRNA stability through m6A demethylation activity [130]. These findings suggest that ALKBH5 may exert cell source-specific regulatory effects on MSC senescence, depending on the target genes and the m6A landscape. m6A readers include YTHDF1/2/3, YTHDC1/2, eIF3, HNRNP and IGF2BP1/2/3, which determine the function of m6A on RNAs at multiple levels. YTHDF1 promoted osteogenesis of BM-MSCs through regulating translation of ZNF839 [131], while YTHDF1 knockout impaired osteogenesis of BM-MSCs in vitro and resulted in decreased bone mass in vivo [131]. YTHDF1 also enhanced chondrogenesis by activating the Wnt/β-catenin signaling pathway [132]. WTAP-YTHDF1-mediated m6A modification of IDO1, PD-L1, ICAM1, and VCAM1 mRNAs contributed to the regulation of IFN-γ–licensed immunosuppressive functions in MSCs [133]. YTHDF3 stabilized IL32 mRNA and promoted the osteogenic differentiation of AS-BMSCs through an m6A-dependent mechanism [134]. While these findings highlight the importance of m⁶A in MSC stemness and function, the precise regulatory mechanisms remain to be fully elucidated.

## NON-coding RNAs in MSC stemness

Noncoding RNAs (ncRNAs), including miRNAs, long non-coding RNAs and circular RNAs, are functional RNA molecules that are not translated into protein. Studies have shown that ncRNAs play a critical role in maintaining MSC stemness.

### miRNA

The senescence of MSCs during in vitro expansion greatly hampers their clinical applications. The molecular mechanisms regulating the senescence of MSCs remain poorly understood. MicroRNAs (miRNAs) have recently been shown to act as key regulators of cell fate decisions, including self-renewal, differentiation, senescence, and apoptosis [135]. miRNAs maintain MSC homeostasis and modulate their therapeutic potential by precisely regulating gene expression and key signaling pathways via posttranscriptional regulation. They primarily function by binding to the 5’ or 3’ untranslated regions (UTRs) of target mRNAs, inhibiting translation or promoting mRNA destabilization through canonical and non-canonical pathways, thereby leading to post-transcriptional gene silencing.

**Fig. 4:**
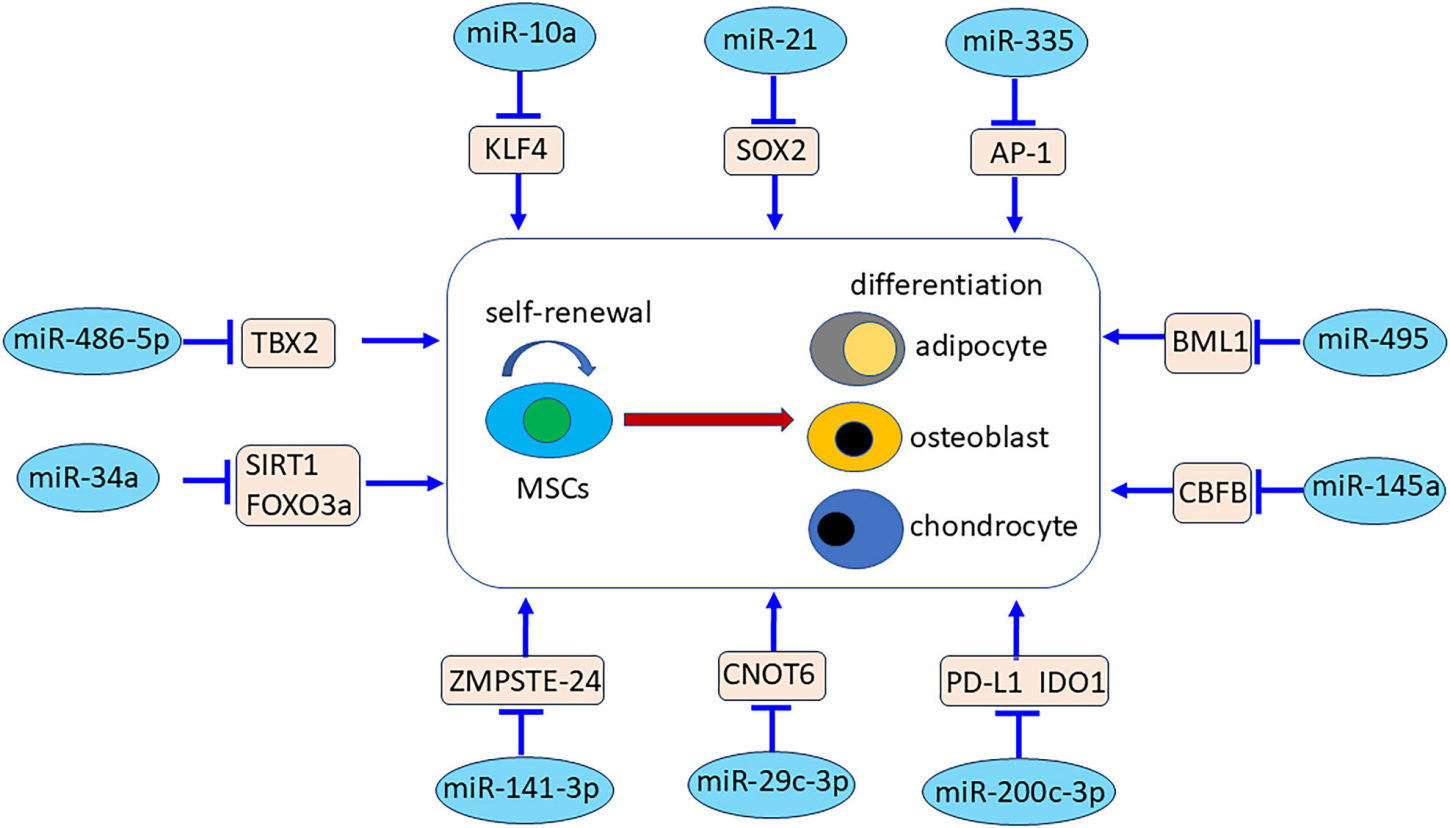
miRNAs regulate MSC stemness via different target genes. miRNAs play critical roles in maintaining stemness and determining the fate of MSCs as regulatory signals. miR-10a decreases cell senescence and improves three lineages of differentiation by repressing KLF4. miR-21 overexpression reduces clonogenic and proliferative potential, slows down adipogenesis and chondrogenesis, and improves osteogenesis by inhibiting SOX2. miR-335 overexpression results in early senescence of hMSCs, abolishes in vivo osteo-chondro potential of hMSCs, and deactivates their immunomodulatory capacity through inhibiting AP-1 activity. miR-495 induces senescence of MSCs via BMI1. miR-145a slows down osteogenesis by targeting core binding factor beta (Cbfb). miR-200c-3p interferes with immunomodulatory capacity by modulating PD-L1 and IDO1 expression of MSCs. The overexpression of miR-29c-3p promotes the senescence of MSCs, decreases proliferation, and slows down osteogenic differentiation by targeting CNOT6. miR-141-3p upregulates prelamin A and results in cellular senescence of hMSCs by decreasing ZMPSTE24 expression. Overexpression of miR-34a increases apoptosis and senescence, impairs the cell vitality of MSCs via the SIRT1/FOXO3a pathway. miR-486-5p inhibits TBX2, resulting in the upregulation of p21, to suppress mitotic clonal expansion and adipogenesis of MSCs.

miRNAs regulate MSC differentiation into multiple lineages by targeting transcription factors and signaling molecules. Senescence-associated miRNAs modulate aging-related pathways in MSCs, with their dysregulation contributing to diminished stemness and compromised regenerative capacity in aged populations. miR-10a levels are significantly reduced in aged hBM-MSCs. Upregulation of miR-10a increased three lineages of differentiation, and reduced cell senescence, whereas miR-10a knockdown produced opposite effects by repressing KLF4 [136]. In vivo, the transplant of miR-10a overexpressing aging MSCs improved heart function by activating Akt and stimulating the expression of angiogenic factors [137]. miR-21 was expressed at higher levels in round-shaped (RS)- amniotic fluid (AF)-MSCs and BM-MSCs compared with spindle-shaped (SS)-AF-MSCs. The miR-21 overexpression decreased SOX2 expression in SS-AF-MSCs, leading to reduced proliferation, clonogenicity, and cell cycle arrest. In differentiation, miR-21 overexpression improved osteogenesis and slowed down adipogenic and chondrogenic differentiation in SS-AF-MSCs. These suggested that miR-21 regulates MSC stemness by regulating Sox2 expression in human MSCs [135] (Fig. 4). miR-335 significantly increased during in vitro expansion of MSCs, and its overexpression led to early senescence of hMSCs, and abolished in vivo osteo-chondro potential of hMSCs and impaired their immunomodulation through inhibition of AP-1 activity [138]. Overexpressing miR-495 induced cell cycle arrest in S phase and promoted cell apoptosis. Molecular mechanism studies revealed miR-495 induced senescence of MSCs via Bmi-1 [139]. p53 is upregulated during MSC senescence and osteogenesis, correlating with increased expression of miR-145a, which is induced by p53. Overexpression of miR-145a promoted senescence and inhibits osteogenesis in MSCs. Mechanism studies showed that p53-induced miR-145a inhibited osteogenesis by targeting core binding factor beta (Cbfb) [140]. miR-200c-3p increased at the late passage of AD-MSCs, its overexpression promoted MSC proliferation and prevented senescence in late passaged AD-MSCs, whereas its knockdown produced opposite effects. Hypoxic preconditioning decreased the senescence of olfactory mucosa MSCs (OM-MSCs) in vitro by upregulating autophagy through the PI3K signaling pathway via targeting PTBP1. Upon transplant, hypoxic preconditioning enhanced survival and tissue-protective capability of OM-MSC in vivo. Hypoxia significantly increased miR-326 expression, overexpression of miR-326 mitigated OM-MSC senescence under normoxia, whereas knockdown of miR-326 increased OM-MSC senescence under hypoxia [141]. The miR-29c-3p expression significantly increased during the MSC senescence. The overexpression of miR-29c-3p promoted senescence of MSCs, decreased proliferation and slowed down osteogenic differentiation whereas miR-29c-3p knockdown produced the opposite effects. Luciferase reporter assays showed that miR-29c-3p affected MSCs by targeting CNOT6 through p53-p21 and p16-pRB pathways [142]. miR-141-3p is overexpressed during MSC senescence, leading to decreased ZMPSTE24 expression and subsequent accumulation of prelamin A in hMSCs. The buildup of prelamin A at the nuclear envelope contributed to cellular senescence. Therefore, microRNA-141-3p plays a role in MSC aging by directly targeting ZMPSTE24 [143]. miR-34a targeted by TP53 plays roles in pro-senescence and pro-apoptosis of MSCs via SIRT1/FOXO3a pathway. Overexpression of miR-34a significantly increased apoptosis and senescence, and impaired the vitality of MSCs [144]. miR-486-5p suppressed mitotic clonal expansion and adipogenesis by inhibiting TBX2 to upregulate p21 [145] (Fig. 4). miRNAs also influence the immunomodulatory function of MSCs. MiR-200c-3p modulated PD-L1 and IDO1 expression and interfered with immunomodulatory capacity of AD-MSCs [146] (Fig. 4). These data show that miRNAs serve as critical molecular switches in MSC biology, orchestrating complex gene networks that govern stem cell fate and function. Understanding and harnessing miRNA-mediated regulation offers new opportunities to enhance MSC-based therapies through targeted genetic or pharmacological approaches.

### Long non-coding RNAs

Long-noncoding RNAs (lncRNA) are a group of multifunctional, non-protein-coding RNAs with lengths of more than 200 nucleotides, which participate in chromatin imprinting, splicing, remodeling, transcriptional regulation, and translation, thereby governing fundamental biochemical and cellular biology processes, including MSC stemness. lncRNA HOTAIR regulated proliferation and differentiation of MSCs. DNA methylation at various CpG sites at the HOTAIR locus increased significantly during the expansion of MSCs. HOTAIR overexpression decreased adipogenesis of MSCs, whereas HOTAIR knockdown impaired proliferation and improved adipogenesis of MSCs. Perturbed HOTAIR changed DNA methylation profile at HOTAIR binding sites, suggesting HOTAIR targets specific sites in the genome through triple helix formation [147]. lncTCF7 activated the Wnt signaling pathway essential for maintaining MSCs in a stem-like state [148]. miR-146a suppressed the proliferation of BM-MSCs partly through inhibiting lncRNA EPB41L4A-AS1 and SNHG7 [149]. lncRNA H19 increased the proliferation, migration, and differentiation of hDP-MSCs via EZH2-dependent LATS1 at H3K27me3 [150]. lncRNA LINC01255 regulated the senescence of hMSCs by inhibiting the transcription of MCP-1 [151]. Beyond those roles, lncRNAs contain small open reading frames (smORFs), which code for microproteins that may fulfill a functional role in MSC biology as post-translational modifiers. Using ribosome profiling (Ribo-seq), 15 lncRNA with 35 smORF were identified in hAD-MSCs [152]. lncRNA RAD51-AS1 levels in hBM-MSCs from osteoporosis patients were significantly lower than those in healthy donors. Knockdown of RAD51-AS1 significantly decreased the proliferation and osteogenesis of hBM-MSCs while increasing their apoptosis. Conversely, overexpression of RAD51-AS1 significantly increased the proliferation, in vitro osteogenesis and ectopic bone formation of hBM-MSCs. Mechanistically, RAD51-AS1 binds to YBX to inhibit the translation of Smad7 and Smurf2, thereby increasing PCNA and SIVA1 [153].

### Circular RNAs

Circular RNAs (circRNAs) are a novel class of noncoding RNAs generated post-transcriptionally from protein-coding genes through back-splicing of pre-mRNAs. This process covalently links the 3’ splice acceptor site to the 5’ splice donor site via a phosphodiester bond, resulting in stable circular RNA structures. Due to the adopted closed loop conformation without free 5’ and 3’ ends, circRNAs cannot be degraded by the 3’–5’ exonucleases and are endowed with longer half-lives. Multiple circRNAs have been shown to play pivotal roles in regulating the differentiation and proliferation of MSCs through diverse molecular mechanisms. By high-throughput screenings, circFOXP1 was identified as decreased circRNA during differentiation of MSCs. CircFOXP1 is abundant in undifferentiated MSCs, circFOXP1 silencing greatly slowed down MSC differentiation via direct interaction with miR-17-3p/miR-127-5p, resulting in the change in non-canonical Wnt and EGFR pathways [154]. circRNA CDR1as levels decreased during MSC differentiation. Knockdown of CDR1as reduced proliferation, expression of stemness-associated genes, and differentiation potential in hUC-MSCs [155]. mmu_circRNA_003795 was significantly upregulated by calcitonin gene-related peptide in BM-MSCs. Silencing mmu_circRNA_003795 markedly decreased FOSL2 expression and cell proliferation, increased mmu_miR-504-3p levels, and caused significant alterations in the BM-MSC cell cycle [156]. circFOXP1 levels were significantly decreased in Osteoporosis (OP) patient bone tissues. circFOXP1 enhanced hASC osteogenesis by sponging miR-33a-5p, thereby increasing FOXP1 expression through direct targeting [157]. The expression of circ-016901 increased following irradiation treatment. Silencing of circ-016901 increased the proliferation of BM-MSCs and ameliorated apoptosis induced by irradiation through the miR-1249-5p/HIPK2 axis [158]. Aberrant expression of CircRNAs in MSCs was observed in BM-MSCs from postmenopausal osteoporosis (PMOP) patients [159]. Moreover, CircRNA PVT1 sequestered miRNA-199a-5p, preventing it from inhibiting SIRT1, a negative regulator of senescence, in tendon progenitor MSCs [160]. CircRNA hsa_circ_0006215 promoted osteogenesis of BM-MSCs by binding to miRNA-942-5p, thereby regulating RUNX2 and VEGF [161]. Circular RNA AFF4 enhanced osteogenic differentiation of BM-MSCs by activating the SMAD1/5 pathway via the miR-135a-5p/FNDC5/Irisin axis [162]. Hsa_circ_0066523 promoted proliferation and osteogenesis of BM-MSCs by inhibiting PTEN [163]. Circ_0067680 accelerated osteogenesis of BM-MSCs via the miR-4429/CTNNB1/Wnt/β-catenin pathway [164]. CircStag1 enhanced bone regeneration through interaction with human antigen R (HuR) [165]. CircHGF suppressed proliferation and osteogenesis of BM-MSCs in osteonecrosis of the femoral head by blocking miR-25-3p-mediated targeting of SMAD7 [166]. circRNA422 enhanced osteogenesis of BM-MSCs during early osseointegration via the SP7/LRP5 axis [167]. has_circ_0001485 promoted osteogenesis by activating the TGFβ-BMP signaling pathway through targeting BMPR2 [168]. CircZNF367 inhibited osteogenic differentiation of BM-MSCs by reducing HuR-mediated mRNA stability of LRP5 [169]. circ-Iqsec1 promoted osteogenesis of BM-MSCs via the miR-187-3p/Satb2 signaling pathway [170]. CircSLC8A1 promoted osteogenic differentiation in BM-MSCs through CircSLC8A1/miR-144-3p/RUNX1 pathway [171]. ircRNA hsa_circ_0006859 inhibited osteogenesis of BM-MSCs and exacerbated osteoporosis by downregulating miR-642b-5p and miR-483-3p, leading to upregulation of EFNA2 and DOCK3 [172]. circRNA-ZCCHC14 modulated the chondrogenesis of peripheral blood-derived MSCs through the miR-181a/GREM1 axis [173]. Knockdown of circSERPINE2 delayed MSC senescence, whereas its overexpression accelerated it. Mechanistically, circSERPINE2 regulates MSC senescence via the YBX3/PCNA/p21 axis [174]. In DP-MSCs, circ-FKBP5 inhibited apoptosis and inflammation while promoting osteogenic differentiation via the miR-708-5p/GIT2 axis [175]. circ-FKBP5 enhanced BM-MSC proliferation and osteogenesis by regulating the miR-205-5p/RUNX2 axis [176]. Circ-Plod2 promoted osteogenic differentiation of BM-MSCs by destabilizing Mpo mRNA through interaction with IGF2BP2 [177]. CircCOX6A1 inhibited osteogenesis of BM-MSCs via the miR-512-3p/DYRK2 axis [178]. circNDUFA13 promoted adipogenesis of BM-MSCs by interacting with STAT3 [179]. circSTX12 modulated the osteo-adipogenic balance and proliferation of BM-MSCs through binding to CBL and LMO7 [180]. These findings highlight the regulatory potential of circRNAs in MSC stemness and their promise as therapeutic targets for bone regeneration.

## Mitochondrial function in regulating MSC stemness

Mitochondrial function plays a critical role in regulating the stemness of MSCs. Mitochondrial regulators such as sirtuins play critical roles in MSC homeostasis and differentiation. As the most well-studied sirtuin, SIRT1 promoted mitochondrial biogenesis by deacetylating target proteins and strategies, which plays roles in energy metabolism involved in cell survival and differentiation. SIRT1 activation rejuvenated phenotypes restores decreased cell proliferation of senescent MSCs by inhibiting microRNA-195 to reactivating telomerase [181]. SIRT1 knockout mice displayed defects in defects in tissues from MSCs, such as decreased subcutaneous fat, cortical bone thickness and trabecular volume [182]. SIRT2 inhibited adipogenesis of MSCs by deacetylating FOXO1 [183]. SIRT3, SIRT4, and SIRT5 residing in mitochondria regulated the acetylation landscape of mitochondrial proteins. SIRT3 regulated osteogenesis of MSCs by directly interacting with PGC-1ɑ, which affected binding to the promoters of SOD2. Overexpression of SIRT3 increased osteogenesis of MSCs [184]. SIRT6 positively regulated adipogenesis [185]. SIRT7 decreased during human MSC aging, and SIRT7 deficiency accelerated the senescence of MSCs [186]. NADH dehydrogenase (ubiquinone) iron-sulfur protein 6 (NDUFS6) is a nuclear-encoded mitochondrial protein crucial to the assembly of mitochondrial respiratory complex I (CI). NDUFS6 decreased in aged MSCs, its knockdown by siRNA accelerated BM-MSC ageing, whereas its overexpression rejuvenated senescent cells in NDUFS6−/− BM-MSCs. NDUFS6 knockout enhanced mitochondrial and intracellular reactive oxygen species (ROS) and reduced mitochondrial membrane potential. Mitochondrial ROS inhibitor Mito-TEMPO reverted the process of senescence and decreased the p53/p21 expression of BM-MSCs [187].

Metabolic states play a pivotal role in impacting epigenetic modifications in MSCs by acting through key regulatory enzymes such as Sirtuins and ten-eleven translocation (TET) dioxygenases. These regulators link cellular metabolic states to chromatin remodeling and gene expression, ultimately affecting MSC fate and differentiation potential. Through these pathways, metabolic cues dynamically influence histone acetylation and DNA methylation status, shaping the transcriptional landscape of MSCs. This metabolic-epigenetic interplay is critical for MSC differentiation and has profound implications for regenerative medicine [188]. The balance between glycolysis and oxidative phosphorylation (OXPHOS) plays a crucial role in regulating MSC stemness and differentiation potential. Undifferentiated MSCs tend to rely more heavily on glycolysis for energy production, even in the presence of oxygen, a metabolic state associated with the maintenance of stemness and a low level of reactive oxygen species (ROS). In contrast, a metabolic shift toward OXPHOS is often observed during lineage commitment and differentiation, as mitochondrial activity increases to meet the energy and biosynthetic demands of specialized cell types [189, 190]. Metabolic interventions have emerged as a promising strategy to preserve MSC stemness by modulating key metabolic pathways that influence epigenetic regulation, cellular energy status, and redox balance. By promoting glycolysis over oxidative phosphorylation, it is possible to maintain the undifferentiated state of MSCs and enhance their self-renewal capacity. These interventions help sustain a metabolic profile favorable to stemness, ultimately improving MSC stability and therapeutic potential in regenerative applications.

## Growth factors influencing MSC stemness

Growth factors play an important role in maintaining the stemness of MSCs. These proteins play key roles in various cellular processes, such as self-renewal and differentiation, which are critical for maintaining multipotency and regenerative potential of MSCs. FGF maintains stemness and modulates MSC differentiation, which increases proliferation and clonogenicity, and expression in NANOG, OCT4, and SOX2 of MSCs from human exfoliated deciduous teeth and permanent teeth [191]. FGF also increased stem cell gene expression of MSCs from the apical papilla (SCAP), including STRO-1 and pluripotency genes of NANOG, OCT4, SOX2, and REX1, promoted proliferation and improved differentiation potency [192]. During in vitro osteogenesis, FGF treatment decreased ALPL mRNA levels and enzyme activity while upregulating ANKH mRNA. This led to a reduction in both the Pi/PPi ratio and mineral deposition in stem cells from human exfoliated deciduous teeth (SHEDs) [193]. Treatment of MSCs with FGF-1 and FGF-2 improved adipogenesis, slowed down osteogenesis, and chondrogenesis [194]. HGF and SCF treatment reduced ROS levels, preserved mitochondrial membrane potential, and upregulated mitochondrial-related protein expression in hBM-MSCs. These findings suggest that HGF and SCF help maintain hBM-MSC stemness [195]. IGF1 overexpression increased cell proliferation, migration, and stemness of BM-MSCs, which were more resistant to apoptosis under hypoxia [196].

### Other genes

Glutathione S-transferase theta 1 (GSTT1) was identified as a scalability marker of MSCs. GSTT1-null MSCs demonstrated an advantage in proliferative rate, CFU-F, and telomeres over GSTT1-positive slow-growth MSCs [197]. The transcription factor Frizzled 5 (FZD5) is expressed specifically in highly functional and immature hMSCs, especially in the LNGFR + THY-1 + MSC fraction. FZD5 encodes a transmembrane receptor for Wnt signaling proteins, which contains a conserved extracellular cysteine-rich domain. FZD5 FZD5 knockdown impaired hMSC proliferation and differentiation capacity while accelerating cellular senescence. Conversely, FZD5 overexpression delayed cell cycle arrest and attenuated senescence in MSCs. These results establish FZD5 as a key regulator of MSC stemness and senescence resistance [198]. As a Twist1 target, the transmembrane protein LRRC15 exhibited expression patterns closely correlated with TWIST1. This relationship enables LRRC15 to serve as both a predictive marker for TWIST1-regulated MSC stemness and a sorting tool for high-quality MSCs [199].

## Strategies for maintaining MSC stemness during ex vivo expansion

A key challenge in MSC therapeutics lies in sustaining stemness while maintaining optimal safety and efficacy profiles. Developing reliable strategies to preserve MSC stemness is essential for producing clinically viable cells with consistent therapeutic potential.

### The choice of MSC origin for improved clinical outcomes

MSCs reside in almost all tissues. MSCs derived from distinct tissue sources exhibit significant variations in their functional properties and stemness characteristics. BM-MSCs demonstrate superior chondrogenic and osteogenic differentiation potential compared to AD-MSCs, attributable to their higher expression of TGFβ1 [200–203], whereas AD-MSCs express higher HGF than BM-MSCs [204], corresponding with their enhanced proliferative potential and immunomodulatory capacity [203]. While both BM-MSCs and AD-MSCs similarly inhibit NK cell proliferation, BM-MSCs demonstrate significantly stronger suppression of NK cell cytotoxicity and greater reduction of IFN-γ secretion compared to AD-MSCs [205]. Comparative analysis reveals distinct functional advantages among MSC populations: SHEDs demonstrate superior proliferative capacity, UC-MSCs exhibit optimal immunomodulatory activity, while BM-MSCs show the strongest antigen-presenting potential following IFN-γ stimulation [206]. Skin-MSCs and skeletal muscle-MSCs inhibit a limited adipogenesis. BM-MSCs and AD-MSCs demonstrate comparable characteristics in phenotype maintenance, bioactive factor secretion profiles, and multilineage differentiation potential [207]. BM-MSCs are better than MSCs from human exfoliated deciduous teeth in treating rheumatoid arthritis [208]. Synovial MSCs from patients with osteoarthritis have better clonogenicity, osteogenesis, and adipogenesis, but worse viability and proliferative potential. MSCs from patients with femoroacetabular impingement syndrome display greater chondrogenic potential [209]. Dental tissue (DT)-MSCs proliferate faster, whereas adipose tissue- and gingival-MSCs display higher clonogenicity [210]. UC-MSCs have advantages in tendon differentiation in vitro and regeneration of a full-thickness tendon defect in vivo over BM-MSCs and UC-MSCs [211]. UC-MSCs express higher PGE2 and IL6 than AD-MSCs [204] and have stronger angiogenic capacity than BM-MSCs and AD-MSCs [212]. Compared with BM-MSCs and AD-MSCs, WJ-MSCs proliferate faster [213]. Furthermore, the MSC secretome exhibits significant source-dependent variations in its composition and biological activity [214, 215].

Primary MSCs derived from somatic tissues exhibit limited proliferative capacity and lifespan, restricting their therapeutic scalability. To address this limitation, MSCs can be generated from induced pluripotent stem cells (iPSCs), which offer unlimited self-renewal capacity. Compared to primary MSCs, iPSC-derived MSCs demonstrate enhanced proliferative capacity and extended lifespan, more homogeneous cellular morphology, elevated expression of pluripotency markers with concomitant reduction in mesenchymal markers, HLA class II molecules, VCAM-1 expression. However, these advantages come with functional trade-offs, including relatively immature differentiation potential and reduced immunosuppressive capacity [216]. These data indicate that tissue source should be carefully considered when using MSCs derived from different origins for therapeutic applications.

### Dissecting MSC heterogeneity using scRNA-seq to identify functional biomarkers

To better understand MSC heterogeneity and stemness, various single-cell RNA sequencing (scRNA-seq) studies have been conducted. BM-MSCs comprise both pluripotent-like and multipotent populations. The pluripotent-like population expresses a combination of pluripotency and mesenchymal marker genes, along with elevated levels of glycolysis-related genes that help maintain MSC stemness. In contrast, the multipotent population expresses only mesenchymal marker genes [217]. Various studies have demonstrated that BM-MSCs comprise lineage-specific precursors, including osteogenic, chondrogenic, and adipogenic progenitors, as well as terminal-stage quiescent cells [218]. BM-MSCs consist of distinct subpopulations defined by marker gene expression, including stemness, functional, and proliferative subsets. The stemness subpopulation serves as a progenitor for both the functional and proliferative subpopulations. The functional MSC subpopulation displays enhanced immunoregulatory activity and osteogenic differentiation capacity but exhibits reduced proliferative potential and adipogenic differentiation [219]. Murine BM-MSCs consistently express high levels of genes associated with MSC multipotency across individual cells, along with lineage-specific genes related to differentiation and immunomodulation [220]. Analysis of MSCs derived from multiple tissue sources, including adipose tissue, bone marrow, dermis, and umbilical cord, revealed seven tissue-specific and five conserved subpopulations, each with distinct gene expression signatures. The extracellular matrix (ECM) plays a significant role in contributing to MSC heterogeneity [218, 221]. Both BM-MSCs and WJ-MSCs have been shown to comprise five distinct subpopulations, including specialized stem-like cells with high proliferative capacity and primed mesenchymal progenitor cells (MPCs) predisposed to trilineage differentiation [222]. Single-cell RNA sequencing of MSCs reveals tissue-of-origin-specific differences and inter-donor variability in cell cycle status [223]. scRNA-seq analysis of BM-MSCs suggests that LRRC75A may serve as a valuable biomarker for identifying subpopulations involved in the angiogenic functions of BM-MSCs. A specific MSC subpopulation expressing LRRC75A has been shown to mediate VEGF secretion in response to ischemic conditions [224]. These scRNA-seq transcriptional profiles enabled the identification of distinct MSC subsets and their associated markers, providing a basis for isolating functional subpopulations to enable more targeted and effective therapeutic applications.

### Modulating specific gene expression in MSCs to preserve stemness

Genetic modification holds great potential in improving the functionality and therapeutic outcomes of MSC-based therapy. MSC stemness can be maintained by modulating stemness-related TFs, growth factors, signaling transducers, and stress-related proteins (Table 1). Overexpression of pluripotent genes such as OCT4 [20, 225], NANOG [20, 226], and combination of OCT4 and SOX2 [21] increased proliferation, prevented senescence, and improved differentiation potential of MSCs. Overexpression of growth factors such as FGF2 [227], CTGF [228], and IGF1 [229] increased the proliferation and differentiation potential of MSCs. Overexpression of GR promoted MSC proliferation and osteogenesis [230], whereas overexpression of CD200 increased CFU-F, osteogenesis, and chondrogenesis [231]. Overexpression of HSP20 [232], HSP27 [233], and HSP70 [234] prevented apoptosis and promoted viability. Unlike traditional viral vector-based methods for genetic modification, CRISPR-Cas9 offers two critical safety advantages, including elimination of insertional mutagenesis risks and precise gene editing without dependence on double-strand breaks. This technology maintains genomic integrity while minimizing structural alterations, significantly enhancing its safety profile for clinical applications. Furthermore, CRISPR enables scarless editing and reduces genomic instability, facilitating the development of GMP-compliant MSC lines. In addition, the platform’s capacity for multiplexed gene editing allows simultaneous modification of multiple therapeutic targets, enabling multifaceted MSC engineering, such as enhancing immunomodulatory capacity, tissue homing, or stress resistance. These features establish CRISPR as both a transformative research tool and a clinically superior alternative to conventional genetic modification approaches.

**Table 1:** Effects of genetic modification on MSC stemness.

Group	Target	Source	Delivery approach	KD/KO/OE	Reported molecular mechanism	Stemness-related outcome	Reference
TFs	NANOG or OCT4	hBM-MSCs	Lentivirus	OE	↑cell proliferation genes	↑ proliferation ↑ clonogenicity ↑ chondrogenesis	[20]
	OCT4	Canine AD-MSCs	Lentivirus	OE	↑ CD44, CD73, CD90, CD105	↑ osteogenesis ↑ proliferation	[225]
	NANOG	hAF-MSCs	Retrovirus	OE	↑ OCT4, Sox2 ↑ cell proliferation-related genes	↓ senescence ↑ clonogenicity ↑ proliferation	[226]
	OCT4 + SOX2	hAD-MSCs	Transfection	OE	↑ cyclin D1	↑ adipogenesis ↑ osteogenesis ↑ proliferation	[21]
	NRF2	hBM-MSCs	Adenovirus	OE	↑ SOD-1, SOD-2, HO-1	↑ viability ↓ apoptosis	[283]
	KLF2	hBM-MSCs	Lentivirus	OE	↑OCT4, Nanog, Rex1	↑ proliferation	[36]
Cell cycle regulators	p21	hBM-MSCs	Lentivirus	KD: shRNA	↑ OCT4, Nanog ↑ telomerase activity ↑ telomere length	↓ senescence ↑ proliferation ↑ osteogenesis	[284]
	BMI1	mBM-MSCs	Transgenic mice	OE	↓ ROS ↓ p16, p19	↑ CFU-F ↑ osteogenesis ↑ bone formation	[285]
	pRB	hBM-MSCs	Electroporation or retrovirus	OE	↑ DNMT1 ↓ p16, p21, APO-1	↑ proliferation ↓ senescence ↑ adipogenesis ↑ osteogenesis	[65]
Growth factors	FGF-2	mAD-MSCs	Adenovirus	OE	Nil	↑ proliferation ↑ chondrogenesis	[227]
	FGF-21	mBM-MSCs	Lentivirus	OE	↓ caspase 3, 7	↓ apoptosis	[286]
	CTGF	hBM-MSCs	Transfection	OE	Nil	↑ proliferation ↑ osteogenesis	[228]
	PGF	hBM-MSCs	Transfection	OE	Nil	↑ viability ↑ proliferation	[287]
	IGF1	hBM-MSCs	Adenovirus	OE	Nil	↑ proliferation ↑ chondrogenesis ↑ osteogenesis	[229]
Receptors	Glucocorticoid receptor (GR)	hBM-MSCs	siRNA-loaded PLGA particles	KD: siRNA or antagonist	↑ Telomerase expression ↑ SOD activity	↑ proliferation ↑ osteogenesis ↓ adipogenesis	[230]
	Tissue factor (TF)	hBM-MSCs	Pluronic-based nanocarrier	KD: siRNA	↑ TNF, iNOS, and IL-1β	↑ osteogenesis ↑adipogenesis ↑ paracrine suppression	[288]
	CD200	hBM-MSCs	Transfection	OV	↑ Nanog, OCT4	↑ chondrogenesis ↑ osteogenesis ↑CFU-F	[231]
	Androgen receptor (AR)	mBM-MSCs, mAD-MSCs	KO mice	KO	↑ Akt and ERK1/2 signaling	↑ proliferation ↑ CFU-F	[289]
Telomerase	hTERT	hBM-MSCs	Retrovirus	OE	↑ telomerase activity ↑ telomere length	↓ senescence ↑ osteogenesis	[71, 72]
HSPs	HSP20	rBM-MSCs	Adenovirus	OE	↑ p-Akt ↑ Bcl-2 ↓ Bax ↓ caspase 3	↓ apoptosis ↑ viability	[232]
	HSP27	rBM-MSCs	Lentivirus	OE	↓ caspase 3	↓ apoptosis ↑ viability	[233]
	HSP70	MSCs	Lentivirus	OE	↑ PI3K/Akt pathway	↓ apoptosis ↑ viability	[234]
Hormones	Adrenomedullin (ADM)	rBM-MSCs	Adenovirus	OE	↑ Akt/GSK3β pathway ↑ Bcl-2/Bax ratio	↑ viability ↑ proliferation ↓ apoptosis	[290]
Glycoprotein	Lipocalin 2 (LCN2)	hBM-MSCs	Transfection	OE	↓ p16, p27, p53	↑ proliferation ↓ senescence	[291]
Mitochondrial enzyme	NMNAT3	mBM-MSCs	Cre-LoxP	OE	↑ antioxidant stress ↑ NMNAT3-NAD + -Sirt3 axis	↓ apoptosis	[292]
Other proteins	Alpha-1 antitrypsin (AAT)	hBM-MSCs	Lentivirus	OE	↑ expression of stemness, migration & cell survival genes	↑ self-renewal ↑ differentiation	[293]
	Hepatitis B X-interacting protein (HBXIP)	hBM-MSCs	Transfection	OE	↑ telomerase activity ↑ hTERT, c-Myc, Bcl-2	↑ proliferation	[294]
	Cathepsin K (CTSK)	mBM-MSCs	KO mice	OE	↑ glycolysis	↑ proliferation ↑ osteogenesis	[295]
	Secreted frizzled-related protein 2 (SFRP2)	mBM-MSCs	Retrovirus	OE	↓ nuclear β-catenin ↓ Wnt target genes ↓ Wnt signaling	↑ proliferation	[296]
		hDP-MSCs	Lentivirus, retrovirus	OE		↑ osteogenesis	[297]

### Immortalization

Immortalization allows MSCs to bypass senescence and maintain MSC stemness. MSC immortalization can be achieved by overexpression of hTERT [235–237], c-MYC [238], SV40T [239, 240], hTERT+E6/E7 [241], hTERT knockout+p53KD [242], SV40T+hTERT [243, 244], and Bmi1+hTERT [245, 246]. Immortalized MSCs exhibit significantly extended lifespan while maintaining trilineage differentiation capacity and characteristic MSC surface marker expression. These properties establish them as a sustainable, standardized source for both MSC research and extracellular vesicle (EV) production. However, immortalized MSCs pose non-negligible safety and ethical challenges in clinical settings, especially when derived using viral oncoproteins (SV40T, E6/E7) or cellular oncogenes (c-MYC). A thorough risk assessment of tumorigenic potential, including uncontrolled growth and malignant transformation, is mandatory before clinical translation. Extended in vitro expansion and genetic modification of MSCs carry inherent risks of genomic instability, including chromosomal abnormalities and acquired mutations, which may compromise clinical safety. While immortalized MSCs overcome replicative senescence, they frequently demonstrate altered paracrine secretion patterns, reduced immunomodulatory function, impaired multilineage differentiation capacity. These functional deviations from primary MSCs could substantially diminish their therapeutic potential and safety profile. In addition, the use of viral vectors for gene delivery poses risks such as insertional mutagenesis and dysregulated transgene expression. To address safety concerns in MSC immortalization, current strategies prioritize non-oncogenic approaches, particularly targeted hTERT overexpression and CRISPR-based gene editing. These advanced methods preserve critical MSC functionality while minimizing transformation risks for therapeutic applications. Prior to clinical translation, extensive in vitro and in vivo safety assessments need to be conducted, with strict compliance to GMP, ethical guidelines, and regulatory requirements. Nevertheless, immortalized MSC lines remain a valuable tool for advancing fundamental research in stem cell biology.

### Engineering the MSC niche to preserve stemness

Stem cells, including MSCs, maintain an undifferentiated and self-renewing state through interactions with their microenvironment, known as the stem cell niche. The stem cell pool is preserved by the stem cell niche to provide a pro-quiescent and anti-differentiation environment. It maintains tissue homeostasis by MSC proliferation and differentiation to replenish lost cells following injuries or natural wear and tear. The stem cell niches, including extracellular matrix (ECM), cell-to-cell communication, cytokines, and growth factors, etc, provide structural support as well as appropriate physiochemical, biophysical, and biochemical cues that ultimately govern MSC behavior and fate. The ECM is a three-dimensional network constituted mainly by glycosaminoglycans (GAGs), proteoglycans, and fibrous and adhesive proteins. GAGs are negatively charged polysaccharides, including heparan sulfate (HS), hyaluronan (HA), chondroitin sulfate (CS), and keratan sulfate (KS). Apart from the free polysaccharide of hyaluronan, they are typically sulfated and covalently attached to their respective core proteins to form a larger complex known as the proteoglycan. The long, inflexible chain and hydrophilic nature of GAGs provide a hydrated space within the ECM, thereby equipping the ECM with viscoelasticity and the ability to withstand compressive forces. Multiple studies demonstrate that GAGs are key modulators of signaling pathways in MSCs. HS, which has a high affinity for FGF, forms the co-receptor for FGFRs and REKs. HS stabilizes FGF2-FGF receptor complexes, thereby accelerating the expansion of freshly isolated BM-MSCs and enhancing osteochondral defect repair in preclinical animal models [247]. Similarly, hyaluronan secreted by BM-MSCs helps maintain their osteogenic potential [248].

### Optimized culture conditions to preserve MSC stemness and enhance functionality

The maintenance of MSC stemness is highly sensitive to the culture environment. Suboptimal conditions can lead to early senescence, loss of differentiation potential, and reduced therapeutic efficacy. Compared with serum-containing medium, defined xeno-free medium increased MSC proliferation, CFU-F, and gene expression related to MSC stemness. Notably, immunophenotypes and differentiation potential of expanded MSCs in xeno-free medium retained [249]. However, some studies have reported that MSCs proliferate more rapidly in serum-containing media than serum-free media [250], possibly due to the use of different formulations of serum-free media. Human dental pulp stem cells (DPSCs) cultured in cGMP xeno/serum-free (XSF) medium appeared more elongated and exhibited a decreased proliferation at later passages, along with lower CD105 expression and CFU-F formation compared to those cultured in FBS-containing medium [251]. Compared with 2D culture, 3D spheroid culture improved stemness, differentiation, and regenerative abilities of dental pulp-derived MSCs [252]. Spheroids from hUC-MSCs produced more cells, expressed higher stem cell markers, and had better multipotency [253]. However, spheroid culture also led to reduced MSC viability and proliferation [254]. Dexamethasone priming enhanced the immunosuppressive capacity of UC-MSCs and DP-MSCs to a greater extent than that of AD-MSCs and BM-MSCs [255]. Sodium lactate also plays a crucial role in human MSC stemness. A low concentration of sodium lactate enhanced the stemness of human MSCs through KDM6B-mediated regulation of glycolytic metabolism [256]. Microgravity environment affects stemness of WJ-MSCs, influencing the expression of stemness-related genes, cell proliferation, and apoptosis [257]. Pressure stimuli significantly increased the proliferation of WJ-MSCs [258]. MSCs typically reside in a hypoxic niche in vivo. Hypoxia enhanced proliferation and CFU-F, promoted differentiation and inhibited senescence [259–261], but did not affect immunophenotype [262]. These data show that optimizing culture conditions is critical for preserving MSC stemness and ensuring reproducibility and potency in clinical-grade MSC production. A well-defined, physiologically relevant culture environment supports more consistent therapeutic outcomes.

### Hypoxic environment for enhanced MSC function

The ability to preserve the MSC pool and equip cells with their stemness capability is also highly dependent on the physicochemical properties of the niche, such as oxygen tension. Hypoxia upregulates the expression of hypoxia-inducible factors (HIFs), which are basic helix-loop-helix (bHLH) transcription factors containing a Per-Arnt-Sim (PAS) domain. These factors regulate the expression of various target genes to orchestrate cellular adaptation to hypoxic conditions. HIF-1α activated pyruvate dehydrogenase kinase 1 (PDK1), an inhibitor of pyruvate dehydrogenase (PDH) needed for pyruvate to form acetyl-CoA, thus blocking the entry into the Krebs cycle. By concomitantly overexpressing lactate dehydrogenase A (LDHA), pyruvate will be encouraged to form lactate [263]. Hypoxia maintained MSCs in an undifferentiated and self-renewal state by downregulating p16 and ERK [264] or direct downregulation of E2A-p21 [265]. Hypoxia stimulated the proliferation of MSCs by increasing SOX2, not OCT4 and REX1 [266]. Overexpression of HIF’s glycolytic enzymes and glucose transporters also facilitated metabolic adaption by ensuring sufficient ATP production during hypoxia. The continuous proliferation of MSCs can exhaust their own pool of cells, undergoing error-prone DNA replication. However, severe hypoxia conferred a quiescent immobile phenotype of MSCs through changes in gene expression and spliceosome profile [267]. In addition, hypoxia promoted autophagy of MSCs by upregulating the SCF/c-Kit [268] and ERK1/2 [269] signaling pathways.

### MSC-EVs: pioneering the next generation of therapies

MSCs hold considerable therapeutic promise but face notable limitations in clinical application, including the risk of immune rejection, tumorigenic potential, and stringent regulatory requirements related to the use of live-cell therapies. In contrast, extracellular vesicles derived from MSCs (MSC-EVs) offer a compelling, cell-free alternative that addresses many of these concerns. Compared with MSCs, MSC-EVs are easier to store and handle, exhibit lower immunogenicity, and pose a reduced risk of immune rejection, resulting in enhanced clinical safety. Importantly, MSC-EVs possess the unique ability to cross biological barriers, such as the blood-brain barrier, broadening their therapeutic potential. As nanoscale carriers, they are enriched with a diverse array of bioactive molecules, including proteins, microRNAs (miRNAs), long non-coding RNAs (lncRNAs), and circular RNAs. These components play pivotal roles in intercellular communication and actively regulate signaling pathways involved in tissue repair, immunomodulation, and angiogenesis. Through these paracrine mechanisms, MSC-EVs can replicate many of the therapeutic effects of their parent cells without the associated risks. MSC-EVs have been investigated across a broad spectrum of preclinical and clinical applications, including tissue regeneration, immune-related disorders such as graft-versus-host disease (GvHD), rheumatoid arthritis, and inflammatory bowel disease (IBD), as well as neurological conditions, pulmonary and liver diseases, oncology, anti-aging therapies, dermatological treatments, and even vaccine delivery systems. Taken together, MSC-EVs represent a promising next-generation therapeutic platform that captures the regenerative and immunomodulatory benefits of MSCs while overcoming the limitations of cell-based therapies.

The endosomal sorting complex required for transport (ESCRT) machinery is a major pathway for cargo sorting into exosomes. It comprises four multi-protein complexes that facilitate the recognition, sequestration, and inward budding of cargo-containing vesicles into multivesicular bodies. To maximize therapeutic efficacy, various strategies have been developed to enhance the specificity of EV targeting to desired cell types or tissues. Surface engineering of EVs improves their homing capability by leveraging ligand-receptor interactions, antibody conjugation, and genetic modification of parental cells. Modifying the physiological or environmental conditions of EV-producing cells can also influence EV surface composition and their targeting tropism. Attaching targeting moieties to EV membranes enables precise and controllable modifications without compromising vesicle integrity. Additionally, EVs conjugated with magnetic nanoparticles can be guided to specific sites using external magnetic fields. Incorporation of tissue-specific homing peptides further enhances organ- or tissue-directed delivery. The development of targeted EV delivery strategies holds great promise for precision medicine. Through engineering approaches, EVs can be tailored to achieve enhanced delivery efficiency, specificity, and therapeutic impact across a wide range of diseases.

## Conclusions

MSCs are among the most widely used stem cells in clinical applications due to their regenerative potential, immunomodulatory properties, and multipotency. However, primary MSCs are still disadvantaged by their rarity in the tissues and variation across donors. An alternative unlimited source of MSCs is iPSC-MSCs, which can be derived from iPSCs by stepwise or non-stepwise differentiation protocols [270–273]. Unlike ESCs, iPSCs are reprogrammed from adult somatic cells, thus circumventing the ethical controversy surrounding the destruction of human embryos. This makes iPSC-MSCs a more ethically acceptable source of stem cells. iPSCs possess unlimited self-renewal capacity and pluripotency, providing a continuous source of iPSC-derived MSCs. iPSC-MSCs acquire a rejuvenated status and exhibit a longer lifespan compared to primary MSCs [273–275]. In addition, BM-MSCs exhibit higher expression of immune-related genes, while iPSC-MSCs show elevated expression of proliferation-related genes [216]. iPSC-MSCs display similar cellular morphology, surface antigen, three lineage differentiation, gene expression profile, epigenetic profile, functionality for graft-vs-host disease (GVHD), cartilage, and bone repair [273, 276–279]. iPSC-MSCs have been used in clinical trials to treat GvHD, iPSC-MSCs were safe and well tolerated, no serious adverse events were observed [280, 281], the objective response and complete response rates at day 100 reached 86.7% and 53.3%, respectively. iPSC-MSCs have also shown potential in treating COVID-19, kidney transplants, diabetic foot ulcers, and osteoarthritis.

However, application of iPSC-MSCs raises important ethical considerations that need to be addressed to ensure responsible clinical use. Donor rights, genetic safety, clinical integrity, and equitable access need to be considered. The derivation of iPSCs requires somatic cells from human donors, transparent consent processes must ensure that donors are fully informed about how their cells may be used and that their genetic information is protected under strict privacy regulations. The reprogramming and differentiation processes can introduce genetic mutations or epigenetic abnormalities, this raises concerns about the safety and long-term effects of iPSC-MSCs in clinical applications. Thorough quality control and risk need to be assessed to prevent potential harm to patients. Moreover, rigorous preclinical testing, regulatory oversight, and public accountability need to be enforced to avoid premature clinical application or commercial exploitation of iPSC-MSCs without sufficient evidence of efficacy and safety. Notably, iPSC-MSC-derived EVs have the potential to halt the progression of Sjögren’s syndrome (SS) prior to the onset of sialadenitis [282].

Despite growing interest, the precise molecular mechanisms that maintain MSC stemness remain poorly understood. Key transcription factors and gene networks governing this property have yet to be fully elucidated. Moreover, only a limited number of reliable biomarkers are currently available to assess the stemness and therapeutic potential of MSCs in regenerative medicine. Identifying robust biomarkers using advanced techniques, such as single-cell RNA sequencing (scRNA-seq), is essential to ensure the quality, functionality, and clinical efficacy of MSCs. Therefore, continued research is crucial to fully uncover the mechanisms underlying MSC stemness and to drive the development of safer and more effective MSC-based therapies.

The therapeutic potential of MSCs is fundamentally rooted in their stemness properties; therefore, understanding and controlling these characteristics is key to developing safer, more effective, and predictable therapies. First, a deep understanding of stemness helps avoid the use of over-passaged or aberrantly behaving MSCs that may lose function or gain unwanted characteristics. This is essential for ensuring long-term safety in clinical use. Second, MSCs with preserved stemness show better survival, homing ability, and function in vivo. Understanding how stemness affects these properties helps in engineering or priming MSCs for targeted delivery and sustained activity at injury or inflammation sites. Third, by defining key stemness markers, differentiation capacity, and immunomodulatory functions, researchers can better select high-quality MSCs with consistent therapeutic potential. This improves batch-to-batch consistency and supports regulatory approval. Fourth, Understanding the molecular pathways that govern lineage commitment in MSCs enables precise control over their differentiation into osteogenic, adipogenic, or chondrogenic lineages. This control is crucial for regenerative medicine applications, particularly in repairing bone, cartilage, or soft tissues, such as in the treatment of osteoarthritis. Fifth, knowing the biological cues that maintain or enhance MSC stemness helps in optimizing in vitro expansion protocols and avoiding premature senescence or differentiation during large-scale manufacturing, preserving therapeutic efficacy. Sixth, MSCs’ immunosuppressive effects are linked to their secretory profile and stemness-related pathways. Understanding these mechanisms enables the selection or engineering of MSCs with enhanced anti-inflammatory effects for diseases like GvHD or immune disorders like osteoarthritis. Lastly, stemness influences the composition of MSC secretomes and extracellular vesicles, which are now being explored as cell-free therapeutics. Knowledge of how stemness impacts EV cargo aids in designing more effective off-the-shelf therapies.
